# Intracellular mechanisms of fungal space searching in microenvironments

**DOI:** 10.1073/pnas.1816423116

**Published:** 2019-06-18

**Authors:** Marie Held, Ondřej Kašpar, Clive Edwards, Dan V. Nicolau

**Affiliations:** ^a^Department of Electrical Engineering and Electronics, University of Liverpool, L69 3GJ Liverpool, United Kingdom;; ^b^Department of Bioengineering, Faculty of Engineering, McGill University, Montreal, QC H3A 0C3, Canada;; ^c^Department of Chemical Engineering, University of Chemistry and Technology, Prague, Prague 166 28, Czech Republic;; ^d^School of Biological Sciences, University of Liverpool, L69 7ZB Liverpool, United Kingdom

**Keywords:** fungal growth, Spitzenkörper, microtubules, live-cell imaging, microfluidics

## Abstract

Filamentous fungi that colonize microenvironments, such as animal or plant tissue or soil, must find optimal paths through their habitat, but the biological basis for negotiating growth in constrained environments is unknown. We used time-lapse live-cell imaging of *Neurospora crassa* in microfluidic environments to show how constraining geometries determine the intracellular processes responsible for fungal growth. We found that, if a hypha made contact with obstacles at acute angles, the Spitzenkörper (an assembly of vesicles) moved from the center of the apical dome closer to the obstacle, thus functioning as an internal gyroscope, which preserved the information regarding the initial growth direction. Additionally, the off-axis trajectory of the Spitzenkörper was tracked by microtubules exhibiting “cutting corner” patterns. By contrast, if a hypha made contact with an obstacle at near-orthogonal incidence, the directional memory was lost, due to the temporary collapse of the Spitzenkörper–microtubule system, followed by the formation of two “daughter” hyphae growing in opposite directions along the contour of the obstacle. Finally, a hypha passing a lateral opening in constraining channels continued to grow unperturbed, but a daughter hypha gradually branched into the opening and formed its own Spitzenkörper–microtubule system. These observations suggest that the Spitzenkörper–microtubule system is responsible for efficient space partitioning in microenvironments, but, in its absence during constraint-induced apical splitting and lateral branching, the directional memory is lost, and growth is driven solely by the isotropic turgor pressure. These results further our understanding of fungal growth in microenvironments relevant to environmental, industrial, and medical applications.

## Significance

Many filamentous fungi colonizing animal or plant tissue, waste matter, or soil must find optimal paths through the constraining geometries of their microenvironment. Imaging of live fungal growth in custom-built microfluidics structures revealed the intracellular mechanisms responsible for this remarkable efficiency. In meandering channels, the Spitzenkörper (an assembly of vesicles at the filament tip) acted like a natural gyroscope, conserving the directional memory of growth, while the fungal cytoskeleton organized along the shortest growth path. However, if an obstacle could not be negotiated, the directional memory was lost due to the disappearance of the Spitzenkörper gyroscope. This study can impact diverse environmental, industrial, and medical applications, from fungal pathogenicity in plants and animals to biology-inspired computation.

Filamentous fungi dwell in geometrically, mechanically, and materially heterogeneous habitats, such as animal or plant tissue (1, 2), decaying wood, leaf litter, and soil (3, 4). The ecological ubiquity of filamentous fungi stems, to a large extent, from their remarkable ability to invade, search for nutrients, and thrive within these microenvironments. Because filaments (hyphae) can grow for relatively long distances (millimeters) through media containing no, or low, levels of nutrients, fungal space-searching strategies need to operate independently of chemotaxis (5, 6).

Extensive studies have described the fundamental growth behavior of fungi: For example, hyphal directional growth (7–11), regular branching (12–14), and negative autotropism (15, 16). However, these studies have been performed on flat agar surfaces, in contrast to the 3D, geometrically constrained habitats filamentous fungi naturally encounter.

Advanced fluorescence microscopy studies of fungal growth on nonconstraining open surfaces have revealed several intracellular processes that are essential for hyphal extension and branching (9, 17, 18). First, the positioning of the Spitzenkörper at the hyphal apex correlates with the direction of apical growth and overall cell polarization (19–24). Second, cytoskeleton dynamics (involving microtubules, actin, and motor proteins) mediate the directional, long-distance transport of secretory vesicles from the body of the fungus toward the hyphal apex, carrying materials for building the hyphal cell wall. Whereas microtubule dynamics in fungal growth have been extensively studied (25–30), our understanding of the role of actin filaments is less developed and more recent (31–36). Third, the dynamic process of constructing hyphal walls results in an increase in stiffness from the apex to the base of hyphae (25, 28, 30, 37–40). Finally, concentration gradients of osmolytes (e.g., ions, sugars, and alcohols) (41) along the hypha and between the hyphal cytoplasm and the outside environment produce considerable turgor pressure, which provides a distributed internal driving force for fungal growth that is manifested primarily at the hyphal tip and which enables the fungus to penetrate soft obstacles (17, 42–47).

Microfluidics devices, which have been used to study the behavior of individual bacterial (48–50), mammalian (51, 52), and plant cells (53, 54), and recently fungi (55–57), can be designed to mimic micrometer-sized, naturally constraining habitats. Furthermore, the material of choice for these devices, poly(dimethylsiloxane) (PDMS) (58), is transparent, allowing visualization by microscopy (52, 59), and is permeable to O_2_, allowing in vitro studies in more realistic conditions.

Using advanced microfluidics technology, our previous studies (60–62) with the fungi *Pycnoporus cinnabarinus* and *Neurospora crassa* demonstrated differences in behavior in constraining geometries compared with that on flat surfaces; in particular, fungi grown in a geometrically constrained environment had up to 10 times lower apical extension rates and distances between branches. Translation of the fungal space-searching process into a mathematical formalism (60, 63) revealed that this strategy is analogous to a “master program” with two “slave subroutines”: Directional memory, whereby individual hyphae return to their initial direction of growth after passing an obstacle that forced them to deviate from their course; and obstacle-induced branching, whereby branching occurs only if the hypha encounters an obstacle that totally blocks its growth. “Running” this program results in a significantly deeper exploration of the available space for growth than other possible alternatives (60, 61): That is, turning off either directional memory, obstacle-induced branching, or both subroutines. It was also shown that the fungal space-searching program can find exits in confining mazes quicker than some mathematical algorithms (63). However, these empirical studies do not offer insights into the “hard-wired” intracellular mechanisms underlying the strategy adopted by fungi for efficient searching of their constraining environment.

The roles of the Spitzenkörper, microtubules, and turgor pressure in fungal growth have been studied comprehensively—but only in nonconstraining environments. As the growth behavior of fungi differs considerably between nonconstraining and constraining environments, our present understanding requires refinement. To elucidate containment-induced intracellular processes in fungi, and particularly their role in directional memory and obstacle-induced branching, we used time-lapse laser-scanning confocal microscopy to image the growth of *N. crassa* and the dynamics of fluorescently labeled Spitzenkörper and microtubules in confining microfluidics networks. The results are potentially relevant to various environmental, industrial, and medical concerns, including fungal pathogenicity.

## Results

### Fungal Growth on Flat Agar Surfaces and in Closed Nonconstraining PDMS Geometries.

**Fig. 1.**
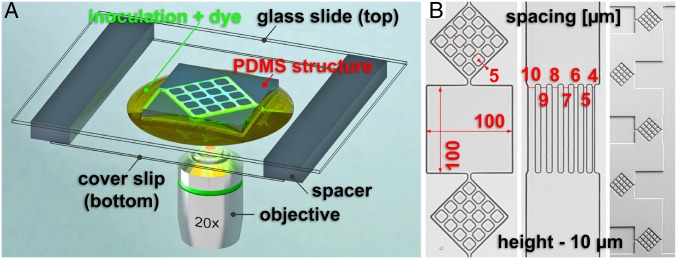
(*A*) Experimental setup for live-cell imaging of fungal growth in microfluidics structures (not to scale). (*B*) PDMS microfluidics structures for confining fungal growth. (*Left*) Three interconnected chambers, of which the middle one was used to investigate nonconstrained growth, while the top and the bottom ones were used to probe lateral branching in constraining environments. (*Middle*) Channels of varying width for probing lateral branching by level of constraint. (*Right*) Overall image of the entry to the chip, probing the response to collisions at acute and near-orthogonal angles, as well as corner responses.

Because the vast majority of reported fungal growth studies have been performed on open agar surfaces, the first step in our study was to establish that the “internal” control in our experiments (that is, using closed, but nonconstraining, large PDMS-made chambers) provided comparable growth conditions with those reported in the literature. Therefore, we performed experiments in closed PDMS microfluidic structures comprising separate chambers (Fig. 1 and *SI Appendix*, Fig. S1) (representative images of fungal growth are presented in *SI Appendix*, Fig. S2), as “internal” control, as well as on agar, as “external” control.

**Fig. 2.**
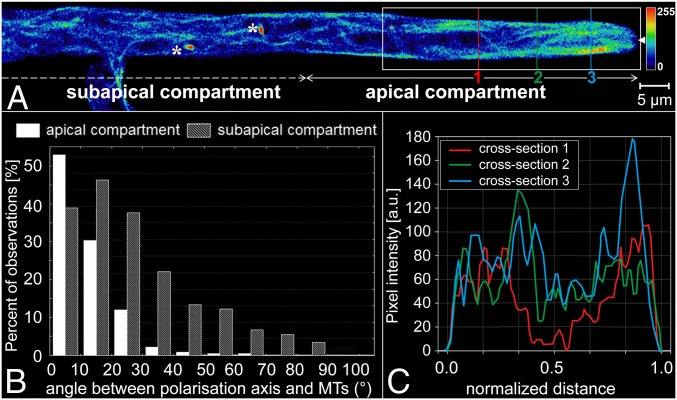
Spatial distribution of microtubules in *Neurospora crassa GFP* in nonconstraining environments. (*A*) Single-plane fluorescence image of GFP-tagged microtubules within a branched hypha. The colors represent the relative spatial density of microtubules (see color map, arbitrary scale, *Right*). The asterisks indicate mitotic spindles, and the solid white arrowhead at the tip indicates the position of the Spitzenkörper. (*B*) Histogram of microtubule (MT) deviation angles from the hyphal polarization axis in the apical and subapical compartments. (*C*) Microtubule density profiles, plotted as fluorescence intensities along the vertical lines (1 to 3) drawn across the hypha in *A*. The hyphal diameter (∼7 µm) was normalized to offset small variations at different sections through the apical compartment.

The comparison of fungal growth on agar (our external control, and published data) and in closed/nonconstraining conditions (our internal control) demonstrates that they elicit similar growth behavior (Fig. 2, Table 1, and *SI Appendix*) (comparison between agar and PDMS in *SI Appendix*, Table S1 and Fig. S3). First, the cross-sectional apical profiles of the hyphae were parabolic and symmetrical (Fig. 2*A* for internal; and *SI Appendix*, Fig. S4 for external control). Second, the Spitzenkörper was centered at the hyphal apex (*SI Appendix*, Fig. S5 and Movie S1), with small periodic oscillations perpendicular to the growth direction (Movie S2). Third, the microtubules were longer and less aligned with the hyphal axis when further away from the hyphal apex (Movies S3 and S4). This is seen as a broadening of the distribution of the deviations of microtubule angles from the hyphal axis (histograms in Fig. 2*B* representing *n* = 852 microtubules in 20 hyphae, for internal control; and *SI Appendix*, Figs. S4 and S6 for external control). Furthermore, the lateral distribution of microtubules indicated that, while they populated both cortical and central cytoplasmic regions (the entire width of the hypha), their density was higher in the cortical region (Fig. 2*C* for internal control; *SI Appendix*, Fig. S7 for external control) (*SI Appendix*, Table S2 and Fig. S8 present a statistical comparison between the controls). The microtubules extended into the apical dome, displaying a characteristic microtubule-depleted zone in the distal central region that colocalized with the Spitzenkörper (Movie S3). Long-term imaging (5 to 10 min) showed that microtubules occasionally traversed the Spitzenkörper position and frequently terminated at the apical cell wall. The estimated microtubule polymerization rate was 26.4 ± 8.6 µm⋅s^−1^ (*n* = 412 measurements from 98 microtubules). Finally, long-term imaging showed that this organization along the hyphal axis is interrupted when microtubules passed a septum (*SI Appendix*, Fig. S9 and Movie S5).

**Table 1.** Comparison of intracellular processes involved in the growth and branching of *N. crassa* in open and constraining environments

Growth	Hypha	Spitzenkörper	Microtubules
Nonconstraining geometries			
Single hypha	Profile: Parabolic, laterally symmetrical	Location and dynamics: Central, at the hyphal apex; permanently present	Orientation: Parallel to axis Distribution: Axially symmetrical Dynamics: Population relatively constant
	Source: Agar (65–67)* and CNC*	Source: Agar (68)* and CNC*	Source: Agar (43–45)* and CNC*
			
Lateral branching	Occurrence: Statistically regular Angle: ∼45° Profiles: Parabolic for parental, daughter hyphae Apical extension: Reduced during branching	Location and dynamics: Central, at the hyphal apices; permanently present in parental hypha; early appearance in the daughter hypha	Orientation: Parallel to hyphal axes Distribution: Axially symmetrical Dynamics: Population relatively constant
	Source: Agar (21)* and CNC*	Source: Agar (21) and CNC*	Source: Agar (21) and CNC*
			
Apical branching	Occurrence: Regular, but rare Angle: V-shaped, ∼45° Profiles: Initial round-up for the twin hyphae Apical extension: Reduced during branching	Location and dynamics: It retracts from the apex and disappears; then, two Spitzenkörper centers emerge at the centers of hyphal apices	Orientation: Parallel to hyphal axes Distribution: Axially symmetrical Dynamics: Population relatively constant
	Source: Agar (21)	Source: Agar (21)	Source: Agar (44, 74)
			
Constraining geometries			
Nestling	Occurrence: Triggered by contact at acute angles Angle: Change of direction as dictated by the wall Profiles: Skewed off-axis, toward the wall Apical extension: Unchanged	Location and dynamics: Off-axis location, pressing against the obstacle; return to central position after passing the obstacle	Orientation: Aligned off-axis Distribution: Axially asymmetrical, “cutting corners” Dynamics: Population relatively constant
			
Hit & split	Occurrence: Triggered by near-orthogonal collisions Angle: T-shaped, at ∼180° Profiles: Triangular; then, progressively parabolic Apical extension: Constant during splitting	Location and dynamics: It disappears during splitting of parental hypha; then, two Spitzenkörper centers form centrally at the apex of twin branches	Orientation: Random close to the splitting Distribution: Random close to the splitting Dynamics: Substantial dissolution; then, formation in twin hyphae
			
Branching in/after tightly constraining channels	Occurrence: Triggered by free space for branching Angle: Dictated by geometry Profiles: Parabolic for parental hypha; circular, then increasingly parabolic for daughter hypha Apical extension: Constant during branching	Location and dynamics: Parental Spitzenkörper progresses unchanged; the daughter hypha forms its own Spitzenkörper early and centrally	Orientation: Parallel to the hyphal axes Distribution: Axially symmetrical Dynamics: Populations relatively constant

CNC, confined, but nonconstraining.

* Present study.

The lateral branching behavior (branching at ∼45° with movement of microtubules into the daughter hypha) was also similar on agar and in closed/nonconstraining PDMS chambers (*SI Appendix*, Figs. S10 and S11 and Movie S6). The central positions and sizes of the Spitzenkörper were also similar (*SI Appendix*, Figs. S11–S13).

After establishing the experimental equivalence between the external control on agar and the internal control in large PDMS chambers, we investigated the effect of geometrical constrainment on hyphal growth using PDMS structures. The geometry of the microfluidic network (Fig. 1*B*) exposed the hyphae to a high density of various structural features (60, 61), such as corners, channels, and entrances and exits from the chambers. This variety of structural features allowed us to observe the intracellular mechanisms of hyphal growth and branching, grouped in three categories of events: Collision with obstacles at acute angles of approach, frontal collision with obstacles, and growth in tightly constraining geometries.

### Collision with Obstacles at Acute Angles of Approach.

**Fig. 3.**
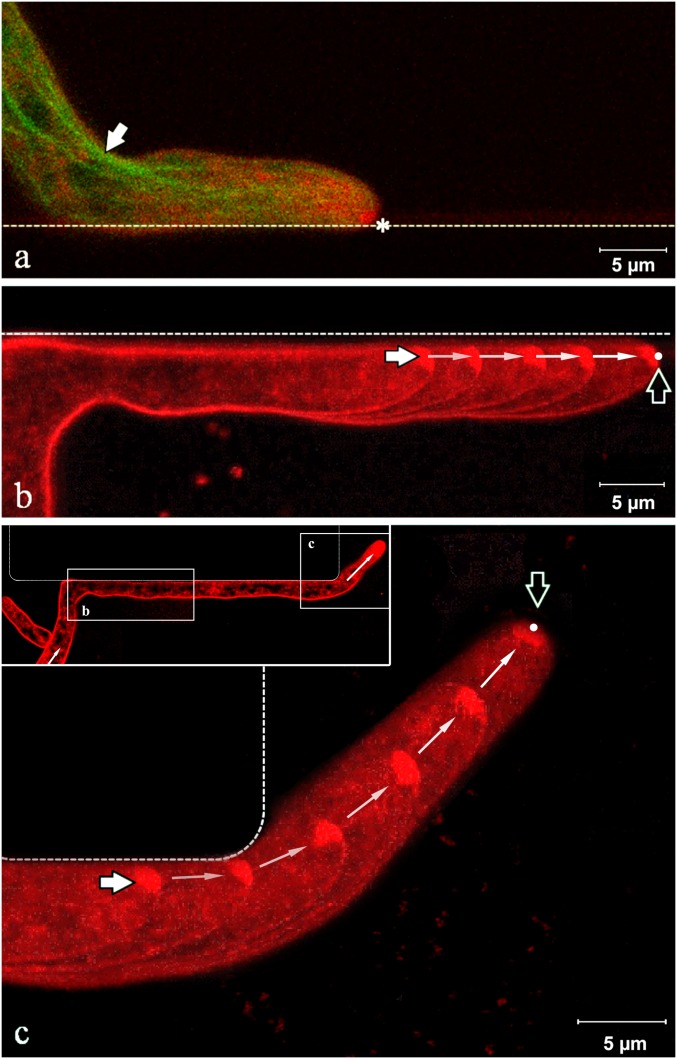
Spitzenkörper and microtubules dynamics in somatic hyphae nestling against a wall. (*A*) Spitzenkörper (labeled with FM4-64, pseudocolored red) and microtubules (genetically tagged with GFP, pseudocolored green) in the apical hyphal region growing along a PDMS wall (dashed line). The parabolic apex profile is skewed toward the wall. The Spitzenkörper (asterisk) is displaced from its usual central position at the apex as growth is obstructed. The microtubules follow the shortest path toward the Spitzenkörper (white arrow) and are displaced from the central median of the hypha. (*B*) Trajectory of the Spitzenkörper along the wall during nestling. The image is an overlay of five snapshots taken over 4 min. The white and black arrows indicate the beginning and the end, respectively, of the Spitzenkörper trajectory. (*C*) Upon reaching the end of the wall, the hypha recovers its symmetrical parabolic profile, and the Spitzenkörper gradually returns to the apical center. The near-orthogonal angle of contact of the hypha with the horizontal wall is the result of shifting the base by the growth of the daughter hypha on the left. The image is an overlay of six snapshots taken over 7.5 min; the white and black arrows indicate the beginning and the end, respectively, of the Spitzenkörper trajectory. The images in *B* and *C* are from the same hypha at different times, as indicated in the *Inset* of *C*. The complete sequence of images is presented in Movie S7.

At acute angles of approach, that is, lower than 35° relative to the fixed obstacle surface, hyphae closely followed the contour of the immobile obstacle, a process previously termed “nestling” (60, 61). To establish the underlying intramolecular mechanisms responsible for nestling, we imaged the growth of the hyphae (*n* = 26) when colliding with PDMS walls at acute angles. We found that nestling dynamics (Fig. 3 and Movie S7) present three phases:

1) Before encountering the wall: Similarly to experiments in nonconstraining geometries, the hyphal profile was symmetrical, with the Spitzenkörper located centrally at the apex and the microtubules distributed symmetrically. We consistently observed the absence of any anticipatory change in behavior even before an imminent contact, suggesting the absence of any sensing mechanism.
2) Nestling: We observed four major changes in hyphal morphology upon encountering a wall. First, the growing hypha followed the constrained path imposed by the obstacle as it slid along the wall in the direction of least deviation (Fig. 3*A* and *SI Appendix*, Fig. S14, *Top*). Second, the longitudinal hyphal cross-section shape lost its symmetry and became considerably skewed toward the wall. The hypha continued its progress in close contact with the wall, maintaining this skewed tip profile. Third, the Spitzenkörper markedly shifted away from its previously central apical location, toward the wall. This displacement persisted over distances at least longer than several hyphal diameters (Fig. 3*B* and *SI Appendix*, Fig. S14, *Bottom*). Skewing of the apex during nestling was constant over time: That is, in nestling events in a sequence of up to 10 chambers. Fourth, microtubules tended to gather near the inside edge of the hyphal bend (white arrow in Fig. 3*A*) and toward the wall at the tip (Fig. 3*A* and *SI Appendix*, Fig. S15). The nestling behavior of the Spitzenkörper (that is, shifting away from the axis toward the wall opposing the initial direction of growth) also occurred when a hypha was able to circumnavigate a small immovable obstacle (*SI Appendix*, Fig. S16).
3) Return to nonconstrained growth: After overpassing the end of the wall, within a distance approximately equal to the hyphal diameter, the hypha quickly recovered its original growth direction. Additionally, the hypha resumed its symmetrical profile; the Spitzenkörper simultaneously returned to a central position (Fig. 3*C* and *SI Appendix*, Fig. S17) (Movie S7 presents the complete time series); and the microtubules recovered their symmetrical transversal distribution. Within the spatial range of observation (spanning 10 chambers, each with a length of 100 µm, and observing more than 100 events), the accuracy in the recovery of the direction of hyphal growth did not diminish over time, having negotiated successive bends through the device, or with increasing distance from the initial branching point of that hypha (*SI Appendix*, Fig. S18).

### Frontal Collision with Obstacles.

**Fig. 4.**
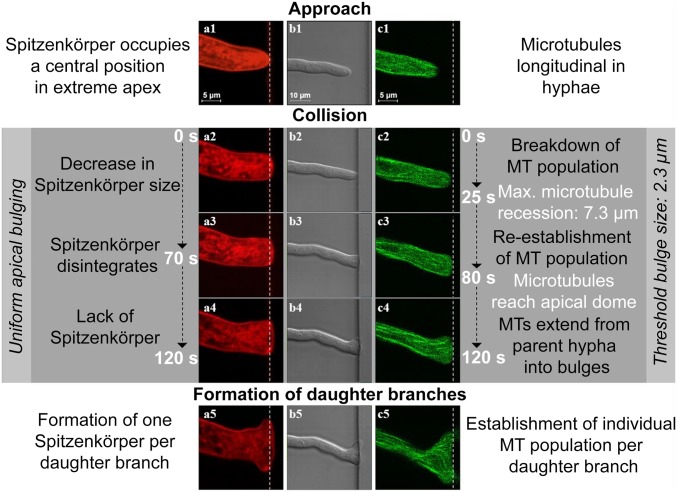
Phases during frontal obstacle-induced nestling branching following collision with a PDMS wall (white dashed lines). Columns *A* and *C* show fluorescence images of the labeled Spitzenkörper (red) and microtubules (green), respectively, and column *B* shows differential interference contrast images of a hypha. The hypha deforms the elastic PDMS slightly from its original position (*B3* and *B4*). During the approach (*A1* and *A2*), the Spitzenkörper is located at the apical center, and the microtubules organize longitudinally (*C1* and *C2*). Following the encounter, the Spitzenkörper shrinks (*A2*) and ultimately disappears (*A3*), and the microtubules temporarily recede from the apical region (*C3* and *C4*). Concomitantly, the apex grows uniformly (*B3* and *B4*). Finally, two new Spitzenkörper structures form in the daughter branches (*A5*), and the microtubules resume their extension toward both apices (*C5*).

Frontal encounters with a wall, at angles of approach greater than 35° relative to the surface of the immovable obstacle, caused the apices of the hyphae to split, a process termed “hit & split.” To establish the underlying intramolecular mechanisms responsible for the hit & split process, we imaged the growth of the hyphae colliding with PDMS walls at near orthogonal angles (*SI Appendix*, Fig. S19). Repeated imaging (*n* = 37 events) provided evidence for a three-phase intracellular process (Fig. 4, *SI Appendix*, Figs. S19–S22, and Movie S8):

1) Polarized approach, before encounter (“Approach” in Fig. 4 *A1*, *B1*, and *C1*): If a hypha approached a wall, similarly to the prenestling phase, microtubules were oriented longitudinally, terminating at the apical region of the cell (*SI Appendix*, Fig. S20*A*).
2) From the moment of encounter to branching (“Collision” in Fig. 4 *A2*–*A4*, *B2*–*B4*, and *C2*–*C4*) comprised three stages: In stage 1 (Fig. 4 *A2*, *B2*, and *C2*), the immovable obstacle blocked the hypha in the direction of growth, causing a small deformation in the elastic PDMS wall (*SI Appendix*, Figs. S19*C* and S21*A*). Hyphal growth then continued quasiorthogonally to the polarization axis, resulting in lateral bulging in the apical region. Simultaneously, the microtubules depolymerized, and the filament ends receded rapidly from the apex (Fig. 4*C2* and *SI Appendix*, Fig. S20*B*). At 25 ± 13 s after the collision, the average distance between the obstacle and the microtubule receding end was 7.3 ± 3.7 µm. The Spitzenkörper shrank gradually but did not retract longitudinally from the apical dome (Fig. 4*A2* and *SI Appendix*, Fig. S21*B*). In stage 2 (Fig. 4 *A3*, *B3*, and *C3*), the hyphal profile continued to develop into two bulges. Total dissolution of the Spitzenkörper occurred toward the end of this stage: That is, 70 ± 40 s after the initial encounter (Fig. 4*A3* and *SI Appendix*, Fig. S21*C*). Importantly, the disappearance of the Spitzenkörper also occurred if the hypha pressed and then penetrated a PDMS wall (Movie S9). The microtubules resumed their extension toward the apex, and, after 80 ± 36 s from the collision, their population appeared to be fully recovered in the hyphae (Fig. 4*C3* and *SI Appendix*, Fig. S20*C*). In stage 3, just before branching was initiated and when the hypha did not have a Spitzenkörper, the uniform apical extension continued laterally, following the constraining geometry. The microtubules again extended to the extreme apical cell walls and migrated from the parent hypha into the nascent bulges, ultimately resulting in an extension along the obstacle walls (Fig. 4*C4* and *SI Appendix*, Fig. S20*D*).
3) Branching (“Formation of daughter branches” in Fig. 4 *A5*, *B5*, and *C5*): Approximately 2 min after the encounter, the uniform extension changed to a bidirectional, polarized pattern, with the bulges reaching 2.3 ± 1.3 µm in length. The sizes of the bulges immediately before forming new branches correlated moderately (*r* = 0.65, *P* < 0.05) with the initial diameter of the parent hypha. The change in polarization pattern coincided with the nucleation of two smaller “daughter” Spitzenkörper structures—one for each new branch (Fig. 4*A*5) (*SI Appendix*, Figs. S21*D* and S22 present the overlap of Spitzenkörper trajectory during the process of hit & split). Independent microtubule populations developed within each branch to conclude the branching process (Fig. 4*C5* and *SI Appendix*, Fig. S20*D*).

Additional evidence of the intracellular processes during the hit & split in more complex geometries is presented in *SI Appendix*, Fig. S23, which shows a sequence of images showing the Spitzenkörper in the process of a hypha colliding with an obstacle, slightly larger than its diameter, which split it into two branches. *SI Appendix*, Figs. S24 and S25 and Movie S10 present the evolution of the microtubules when a hypha collided near orthogonally with a short obstacle that blocked the formation of a second branch. In this instance, once the branch is formed, the microtubules present the characteristic corner-cutting pattern (*SI Appendix*, Fig. S25). Finally, Movie S11 presents a similar lateral branching due to the collision of a hypha with a corner that does not allow the formation of two branches.

### Growth and Branching in Tightly Constraining Geometries.

To establish the underlying intramolecular mechanisms responsible for growth and branching in tightly constrained geometries, we imaged the evolution of the hyphae in channels with widths smaller than their diameter, without and with lateral opening, and in dead-end corners.

First, when *N. crassa* progressed in long, linear, tight channels without lateral exits (*n* = 14), the hyphae branched immediately upon cessation of the confinement: For example, at a channel opening into a larger volume (Movie S12), with both hyphae generating their own Spitzenkörper soon after exit (*SI Appendix*, Fig. S26). Importantly, the behavior manifested during nestling (that is, preservation of the initial direction of growth by the Spitzenkörper before entering the tight channel) was also present (*SI Appendix*, Fig. S27). Additionally, the microtubules exhibited the same pattern: That is, pressing against the wall opposite to the initial direction of growth (*SI Appendix*, Figs. S27 and S28).

**Fig. 5.**
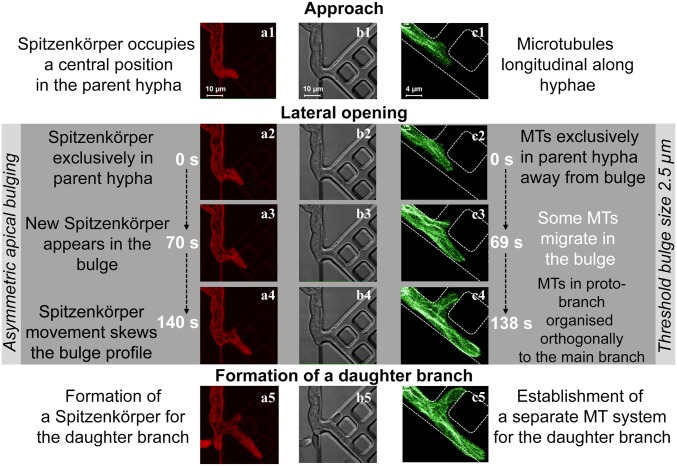
Phases of hyphal branching into a lateral channel (white dashed lines). Columns *A* and *C* show fluorescence images of the labeled Spitzenkörper (red) and microtubules (green), and column *B* shows differential interference contrast images of a hypha. The parent branch preserves its Spitzenkörper throughout. Upon entering the channel (*A1*, *B1*, and *C1*), the Spitzenkörper preserves the initial growth direction (*Top Left* in *A1*), being positioned along the wall. The parent hypha in images (*C1* and *C2*) passes the intersection while the daughter branch forms orthogonally. Whereas the cell wall partially follows the lateral gap (*A2*, *B2*, and *C2*), the formation of the daughter hyphae is delayed by the formation of the Spitzenkörper–microtubule system. Eventually, the daughter hypha forms its Spitzenkörper and microtubule population approximately simultaneously (*A3*, *B3*, and *C3*). Microtubules are initially distributed longitudinally in the parent hypha and do not extend into the bulge. Between frames *C3* and *C4*, the microtubules start to extend from the parent hypha into the bulge, indicating the formation of the daughter hypha. The development of this branch is completed by the formation of an independent microtubule population (*C5*).

Second, for hyphae growing in channels with lateral exits (*n* = 25), branching occurred almost immediately when passing this opening (Fig. 5 and *SI Appendix*, Fig. S29 for Spitzenkörper; *SI Appendix*, Fig. S30 for microtubules; and Movie S13).

The growth and branching into lateral openings proceeded in three phases (*n* = 20 hyphae):

1) Entry and apical growth in the channel (“Approach” in Fig. 5 *A1*, *B1*, and *C1*): Upon entering the confining channel (Fig. 5 *A1* and *B1*), the hypha grew along its initial direction, without turning into lateral channels. Similarly to nestling, the Spitzenkörper was closer to the walls opposite to the initial direction of growth (*SI Appendix*, Fig. S29). The microtubules were oriented longitudinally within the parent hypha (Fig. 5*C1* and *SI Appendix*, Fig. S30*A2*).
2) Formation of a proto-branch (“Lateral opening” in Fig. 5 *A2*–*A4*, *B*2–*B4*, and *C2*–*C4*): If the hypha encountered a lateral opening, the subapical region extended into it, producing a bulge (Fig. 5 *A2*, *B2*, and *C2* and *SI Appendix*, Fig. S30*A1*). The longitudinal orientation of the microtubules in the parent hypha was conserved (without moving toward the bulge, even after the hyphal apex passed the lateral opening), but eventually polarization occurred (Fig. 5*C3* and *SI Appendix*, Fig. S30*B*), followed by microtubule transfer from the parent into the developing branch (Fig. 5*C4* and *SI Appendix*, Fig. S30*C*). Approximately halfway through this process (∼70 s) (Fig. 5 *A3*, *B3*, and *C3*), the emerging branch formed its own Spitzenkörper, and the microtubule populated the branch (*SI Appendix*, Fig. S30*D*).
3) Development of a stand-alone branch (Fig. 5 *A5*, *B5*, and *C5*): Subsequent development was characterized by the formation of a separate population of microtubules and an independent daughter hypha (Fig. 5*C5* and *SI Appendix*, Fig. S30 *E* and *F1*). Interestingly, features associated with directional memory appeared early: For example, the ability of microtubules to cut corners (Fig. 5*C5*). This process occurred within a few minutes of the initial crossing by the parent apex.

**Fig. 6.**
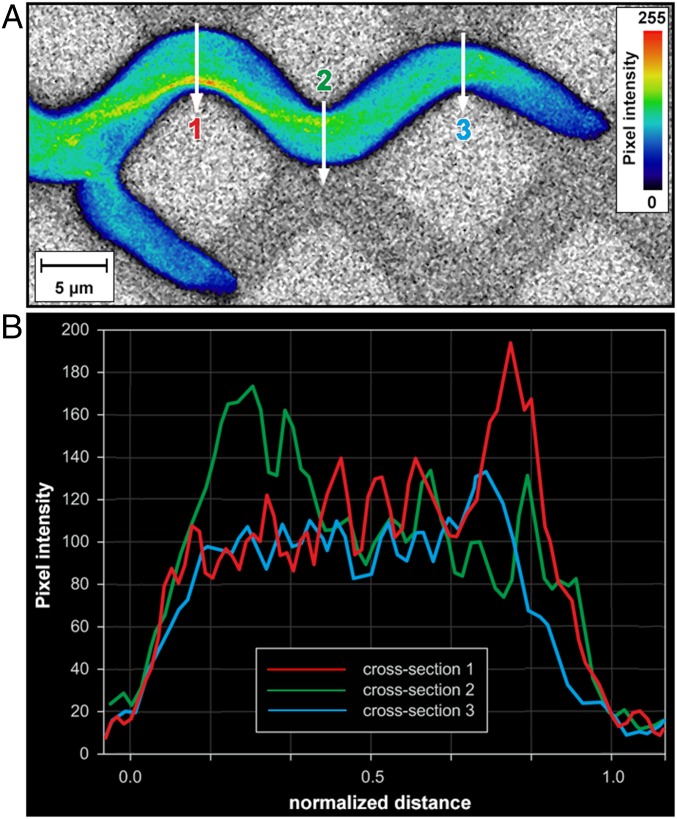
Spatial distribution of microtubules in *Neurospora crassa GFP* in constraining meandered channels. (*A*) Single-plane fluorescence image of GFP-tagged microtubules. The microtubule alignment largely follows the initial direction of growth at the entry into the constraining channel. The colors represent the relative spatial density of microtubules (see color map, *Right*). (*B*) Microtubule density profiles, plotted as the fluorescence intensities along the vertical lines (1 to 3) drawn across the hyphal cross-section in *A*.

Aside from observing the mechanisms involved in branching, the visualization of hyphae growing in tightly constraining channels offered additional evidence regarding the structuring of the microtubule cytoskeleton following changes of the direction of growth, now obligated by the meandering geometries. Similarly to nestling, the pattern of microtubules preferentially distributed toward the wall opposing the direction of growth (“cutting corners” patterns) was also observed when hyphae navigated meandering channels with widths of 5 µm (Fig. 6, *SI Appendix*, Figs. S31 and S32, and Movies S14 and S15), despite the necessity of passing through centrally located septa (*SI Appendix*, Fig. S33).

## Discussion

Studies describing the intracellular processes involved in fungal hyphal extension and branching predominantly used flat, nonconstraining agar surfaces. Advanced microscopy dictates the use of transparent substrates on which the hyphae grow. However, these experimental frameworks (that is, flat surfaces and transparent media) are dissimilar to the natural habitats of filamentous fungi, environments that comprise constraining geometries, which are expected to interfere with the mechanisms of fungal growth being studied. Our previous studies on the growth of the filamentous fungi *P. cinnabarinus* (60) and *N. crassa* (61, 62) in PDMS microfluidic structures identified two efficient space-searching strategies—directional memory and obstacle-induced branching. Summarizing the results of the fluorescence live imaging of the growth of *N. crassa* in microfluidic networks, presented above, when a hypha was deflected by an immovable obstacle, the Spitzenkörper shifted from its central position in the hyphal apex toward the obstacle opposing the growth and returned to its central position when the mechanical constraint ceased. In these instances, the microtubules followed the trajectory of the Spitzenkörper, resulting in cutting corners patterns. Finally, when the immovable obstacle could not be circumnavigated, the Spitzenkörper–microtubules system in the parent hypha disintegrated, followed by branching which allowed the growth to proceed, and concluded with the creation of independent Spitzenkörper–microtubules systems in the daughter hyphae.

### Intracellular Mechanisms of Growth in Nonconstraining PSMS-Made Environments.

We observed that the behavior of *N. crassa* in nonconstraining PDMS-made environments was similar to that on agar, both observed by us and as reported in the literature. First, in our experiments, the hyphal profile was parabolic and symmetrical (Fig. 2*A* and *SI Appendix*, Fig. S4), as also previously demonstrated and comprehensively described mathematically (64–66). Second, the Spitzenkörper was located centrally at the hyphal apex (*SI Appendix*, Fig. S5 and Movie S1) as described in early classical studies (67). Also, the observed oscillations orthogonal to the growth direction (Movie S2) were consistent with a previous report (8). Third, the microtubules were generally orientated parallel to the longitudinal hyphal axis (Fig. 2*A* and *SI Appendix*, Figs. S6–S8 and Table S2), and their accumulation toward the apical region correlates well with previous observations (25, 27, 28). The observed microtubule polymerization rate (26.4 ± 8.6 µm⋅s^−1^) was consistent with previously reported results obtained for hyphal growth on agar (25).

In conclusion, a high degree of similarity exists between the growth behavior and relevant intracellular processes in closed/nonconstraining PSMS-made microfluidic chambers, and those observed during experiments made on agar, as reported here and in the literature. Therefore, the experiments in large microfluidic chambers are valid benchmarks for assessing the impact of constraint on fungal growth.

### Intracellular Mechanisms Responsible for Directional Memory During Nestling.

In general, the extension of a hypha over a flat surface followed a direction determined at the initial branching point, usually at an angle of ∼45° from the parent hypha. We have previously shown (60, 61) that, in constraining geometries, the growth is forced to change direction due to an immovable obstacle, but, once the hyphae circumnavigate it, they recover their initial direction of growth to within an ∼20° error. This directional memory persists even over distances greater than 10 times the hyphal diameter, regardless of the number of encountered collisions. Interestingly, the directional memory has been demonstrated in both *P. cinnabarinus* (60) and *N. crassa* (61), but not in a cytoskeleton-defective *N. crassa* ro-1 mutant (61). This observation suggests that the cytoskeleton plays a key role in maintaining directional memory in constraining geometries.

Our results in nonconstraining environments (presented here, both on agar and in large PDMS chambers lacking internal obstacles) confirmed previous observations that hyphal growth follows the positions adopted by the Spitzenkörper (8). However, although this observation remains valid if hyphae circumnavigate immobile obstacles by nestling, it requires important qualification. Indeed, if a hypha slid past an immovable barrier at an acute angle of contact, the Spitzenkörper functioned like a gyroscope, maintaining the growth direction that the hypha had before the encounter (Fig. 3, *SI Appendix*, Fig. S14, and Movie S7). One possible explanation for this hitherto unknown phenomenon is that the pressure applied to the hyphal wall due to the mechanical contact with the obstacle results in an intracellular signal that triggers consolidation of the hyphal wall at the zone of contact. This process would require the off-axis positioning of the Spitzenkörper and pressure on the contact point between the hyphal wall and the obstacle (as confirmed by additional experiments, in different settings) (*SI Appendix*, Figs. S16, S17, S27, and S29). Furthermore, the off-axis position of the Spitzenkörper creates a skewed microtubule cytoskeleton, which leads to the characteristic pattern of “cutting corners” (Fig. 3*A*)—especially when the directional memory causes hyphae to negotiate corners in various geometries (*SI Appendix*, Figs. S15, S24, S25, S27, S28, S31, and S32 and Movies S12 and S13). This effect is even more remarkable when considering that the microtubules must pass through narrow septa, which are centrally located on the median line of the hypha (*SI Appendix*, Figs. S9 and S33 and Movie S5) (68, 69). The functional synergy between the gyroscope-like Spitzenkörper and the subsequent preferential positioning of the microtubules along a line approximating the initial direction of hyphal growth appears to constitute the underlying intracellular mechanism for directional memory, which was observed for distances at least one magnitude longer than hyphal diameters (the hyphal trajectories in Movie S7 are longer than 100 µm; and the distances in *SI Appendix*, Fig. S18 are several hundred micrometers).

More detailed experiments regarding the role of F-actin structures—actin rings, patches, and cables (33)—which are more difficult to visualize than microtubules (33, 34), might reveal their potential role in directional memory. However, because actin cables are colocalized near the Spitzenkörper and behind actin rings, it is expected that the role of actin is limited, at least in relation to the long range of directional memory.

### Intracellular Mechanisms Involved in Obstacle-Induced Branching During Hit & Split.

Our previous experiments with *N. crassa* (61) showed that containment in various microfluidic structures, comprising channels with widths similar in size with hyphal diameters, results in a shortened distance between hyphal branching points by a factor of 5 to 10 (the growth rate also decreases 10-fold). We also observed (61) that, immediately after the contact between a hypha and a constraining structure at a near-orthogonal angle, branching occurs at the apex of the hypha. This hit & split branching contrasts the behavior presented by *P. cinnabarinus* (60), which branches at a considerable distance behind the hyphal apex.

#### Similarities and differences between the Spitzenkörper dynamics in collision-triggered hit & split and collision-independent apical branching.

The intracellular mechanisms responsible for the collision-induced behavior mentioned above, as revealed by our experiments, present some similarities with the processes previously shown to take place during collision-free apical branching of *N. crassa* on agar (27, 70). For instance, both the disappearance of the parent Spitzenkörper that we observed after microtubule contraction from the apex region and the nucleation of the two daughter Spitzenkörper centers were also observed in the apical branching of *N. crassa* on agar (70). More specifically, in internally triggered apical branching on agar, the Spitzenkörper retracts 12 s after cytoplasmic contraction from the apex which precedes the branching and disappears after another 47 s; later, 45 s after the start of isotropic, uniform, and slower growth of the parental and daughter hyphae, one Spitzenkörper nucleates, followed by a second ∼7 s later, leading to the establishment of two new branches (70). By comparison, in our observations of hit & split branching (Fig. 4 and *SI Appendix*, Figs. S21 and S22), the Spitzenkörper was not visible until 50 s after hitting the obstacle. Moreover, the decrease we observed in Spitzenkörper size, its subsequent disappearance, and the assembly of two new daughter Spitzenkörper centers away from the parent represent a typical sequence of events that also occurs naturally in apically branching fungi: For example, *Sclerotinia sclerotiorum* (21).

Conversely, our experiments regarding the intracellular mechanisms responsible for the collision-induced behavior also show important differences with respect to the processes during collision-free apical branching of *N. crassa* on agar (70). First, on homogeneous agar substrates, the branching of *N. crassa* hyphae occurs predominantly laterally, not apically (70). In contrast, in hit & split branching in constraining environments, we observed that apical branching was the prevalent process. Second, in the absence of a Spitzenkörper, the apical extension stalls in *S. sclerotiorum* (21) and is notably reduced in *N. crassa* branching apically on agar (70). In contrast, this delay in apical extension was not observed in our experiments with *N. crassa* colliding frontally with a wall. We attribute this difference between hit & split branching and the apical branching in nonconstraining environments to different trigger mechanisms. For example, an apical split can occur on agar a few minutes after the induction of an intracellular process free of external stimuli, whereas the immediate response of *N. crassa* following a frontal collision with an obstacle, as observed in the present study, can be the result of a highly localized in time and space contact-induced signal.

#### Similarities and differences between microtubule dynamics in collision-triggered hit & split and collision-independent apical and lateral branching.

The behaviors of the microtubules in apical and lateral branching on agar are similar (27), but we found that they are markedly different during the hit & split response. In unconstrained apical or lateral branching on agar, the microtubule population is relatively unchanged throughout the branching process whereas a hit & split response appeared to trigger the depolymerization of the microtubules (Fig. 4*C2* and *SI Appendix*, Figs. S20 and S21). Furthermore, if a hypha encountered a corner (Movie S11), the resulting budding branch was not initially populated with microtubules, suggesting that the association of microtubules with the apical cell wall is not a prerequisite for selecting a branching site, as has been observed for lateral branching in nonconstraining environments (27), but which could be alternatively explained by cell wall deformation driven by isotropic turgor pressure.

The role of actin in hit & split branching, as with nestling, is yet to be established. However, as it was shown for two species of yeast (71) and for *N. crassa* (72), actin is not present at the tip of invasive hyphae: That is, those pressing against agar in conditions similar to our experiments (Movies S8, S9, and S11). Consequently, it is reasonable to assume that the contribution of actin to hit & split branching is minimal.

### Overlap of Intracellular Mechanisms of Directional Memory and Obstacle-Induced Branching During Lateral Branching.

We found that the lateral branching that occurs in tightly constraining microfluidic channels was only partly similar to lateral branching in nonconstraining conditions. At the beginning of lateral branching in nonconstraining geometries, we observed the association of cortical microtubules with the cell wall at the location of the developing lateral branch. Upon further extension, the microtubules gathered and bent considerably. The severed ends of microtubules then migrated into the branch and resumed polymerization. These observations are consistent with other studies of lateral branching on flat agar surfaces (27). Importantly, though, in our tightly constraining channels, the original Spitzenkörper remained intact in the parent hypha during lateral branching, and a new Spitzenkörper appeared independently within the daughter branch. This has also been observed in lateral branching in nonconstraining conditions (70).

The most obvious difference between lateral branching in tightly constrained geometries and that on flat surfaces was in the place and frequency of branching. These appeared to be dictated by the availability of lateral space, rather than triggered by internal processes, as appears to be the case in nonconstraining conditions. Moreover, in tight channels, there was a close temporal correlation between the presence of the constraining geometry and the lateral branching, enforced by the axis of the available space (e.g., orthogonal in Fig. 6; also *SI Appendix*, Figs. S29 and S30). Also *N. crassa* branched typically and almost immediately after an exit from a bottleneck (Movie S12) (61). These observations suggest that the isotropic turgor pressure is essential for initiating lateral branching events in tightly constrained environments.

Finally, the branching we observed in constraining environments differed from that on open, flat agar surfaces, involving the same genetically tagged *N. crassa* strain (25). In our study, no cortical microtubules were observed to bend or shatter. Cell wall deformation preceded microtubule extension from the parent hypha into the nascent bud, making it appear the dominant event in the chain leading to branch formation. The bulging of the cell wall into an intersection of channels also preceded the formation of a daughter Spitzenkörper (*SI Appendix*, Fig. S23), suggesting that the nucleation of the Spitzenkörper occurs after the initiation of branching, as opposed to lateral branching on open surfaces (25).

Lateral branching in tightly constraining channels appears to be the result of coupling of the Spitzenkörper–microtubules-controlled directional memory for the growth of the parental hypha, simultaneously with turgor pressure-controlled obstacle-induced branching of the daughter hypha.

### Intracellular Mechanisms of Directional Memory and Obstacle-Induced Branching.

By using time-lapse confocal fluorescence microscopy to observe growth of *N. crassa* in constraining microfluidic environments, we revealed substantial differences in the intracellular processes involved in the fungal search for space for hyphal growth, compared with those manifested in nonconstraining conditions. These differences are presented in Table 1.

Our study shows that the intracellular processes involved in the growth of *N. crassa* in constraining geometries are triggered and modulated by the type of obstacles encountered by hyphae. Of the two important behavioral traits of *N. crassa* in growth-constraining environments (61), directional memory appears to arise from the Spitzenkörper “remembering” the initial direction of growth, pressing against opposing obstacles encountered at an acute angle of attack, and then returning to the initial direction when the blocking obstacle is left behind and contact with the hypha ceases. This gyroscope-like dynamic memory is further stabilized by the structuring of the microtubules in the wake of the trajectory of the Spitzenkörper, resulting in the characteristic corner-cutting feature of the microtubule cytoskeleton in meandering channels. Directional memory, described as a behavioral trait of some fungal species (60, 61), may provide biological advantages for filamentous fungi growing and foraging in geometrically heterogeneous environments. Indeed, stochastic simulations showed that suppressing directional memory in *P. cinnabarinus* (60) increases the probability of hyphae being trapped in a network. Furthermore, an *N. crassa* ro-1 mutant that did not display directional memory presented a considerably lower capacity for exiting complex geometries than the wild-type *N. crassa* (61).

In contrast to the intracellular processes involved in directional memory, the Spitzenkörper–microtubules system does not appear to determine the direction of obstacle-induced branching. Indeed, in hit & split events, both the Spitzenkörper and microtubules are absent at the critical point of apical splitting. The obstacle-induced branching observed in species exhibiting directional memory (60–62) suggests that this behavioral trait also affords biological advantages. Indeed, stochastic simulations (60) have demonstrated that obstacle-induced branching leads to a higher capacity for exiting complex networks, but with a lesser benefit than directional memory. Consequently, it appears that *N. crassa* has evolved intracellular processes responsible for directional memory and obstacle-induced branching, with the former being the main driver for the negotiation of complex networks, and the latter a fallback mechanism when directional memory is turned off during near-orthogonal collisions, or when it cannot operate due to the constraints imposed by tight geometries.

### Perspectives and Further Work.

Aside from revealing fundamental intracellular mechanisms involved in fungal growth, this study may have further impact, or suggest further research, as follows:

- Our PDMS microfluidic devices, in conjunction with advanced microscopy imaging, could be used in fundamental microbiology studies to trigger spatiotemporally precise biomolecular events which are modulated by the cellular interaction with the solid environment—for example, to investigate other elements controlling the fungal growth in confined spaces. Two aspects appear to ask for special attention: The mechanisms responsible for the dissolution of the Spitzenkörper and the associated depolymerization of microtubules in the initial stages of hit & split; and the role of actin structures in the hyphal growth in constrained geometries, in particular when the Spitzenkörper/microtubule system is not present or observable.
- Our devices could be designed more closely to mimic fungal environments, to bring about environmental, industrial, and medical applications, including fungal pathogenicity, which is controlled by the successful negotiation of meandering geometries made of multicellular constructs in animals and plants. For instance, the mechanical strength of PDMS could be adjusted to allow the estimation of the forces applied by fungi in various environments, by the measurement of resultant deformations, as already demonstrated (73, 74). Alternatively, the design of the PDMS structures could mimic the structure of the walls of plant or animal tissue in studies on fungal invasion.
- The confinement imposed on the growth of filamentous fungi could be applicable to biologically driven computation. For instance, it was shown (62) that a genetically engineered, cytoskeleton-defective mutant of *N. crassa* that produces short branches preferentially at 90° can solve orthogonal mazes better than the wild-type strain, which overwhelmingly branch at 45°. Furthermore, as the natural space-searching strategies used by fungi have been demonstrated to be more efficient than some artificial algorithms (63), it is possible to use either wild-type or genetically engineered fungi to attempt solving complex physical networks encoding combinatorial mathematical problems, as proposed (75), and recently demonstrated (76). Alternatively, the nuclear dynamics in *N. crassa* (77) could be “streamlined” in networks mimicking real, complex, transportation webs, thus allowing studies on traffic optimization (77–79). A conceptual framework for doing so has been demonstrated for *Physarum polycephalum* (80).

## Conclusions

Our study of the response of *N. crassa* growth to the geometrical constraints imposed by a PDMS-based microfluidic structure has revealed how the Spitzenkörper–microtubule system is closely linked to directional memory when hyphae encounter obstacles at acute angles of contact. Conversely, if the hyphae collide near-orthogonally with fixed obstacles that block their growth, the temporary absence of the Spitzenkörper–microtubule system results in the loss of directional memory, and growth continues due to ever-present isotropic turgor pressure. Finally, if free space becomes available laterally from tightly constraining channels, the directional memory cannot operate, again leaving turgor pressure responsible for hyphal lateral branching.

These findings can accelerate further studies on the intracellular processes driving fungal growth in confined environments and may have impact on a range of environmental, industrial, and medical applications, from fungal pathogenicity in plants and animals to biologically driven computation.

## Methods

### Microfabrication and Experimental Setup.

The microfluidic network (Fig. 1 and *SI Appendix*, Fig. S1) presents various levels of containment to fungal growth, from tight-constraining in channels with widths smaller than the hyphal diameter (5 to 7 µm) to confined, but nonconstraining chambers (100 × 100 × 10 µm). The design of the microfluidic network allowed the investigation of fungal behavior as influenced by various levels of confinement and constraint (detailed in *SI Appendix*, Fig. S34).

### Fungal Species, Growth Media, and Staining.

*N. crassa* was selected as the model organism because we could benchmark our results regarding growth and branching in microenvironments with a large body knowledge related to open spaces and because many mutants are available for experimental studies. *Neurospora crassa rid (RIP4) mat a his-3+::Pccg-1-Bml+sgfp+* mutant strain (henceforth “*Neurospora crassa GFP*”) [Fungal Genetics Stock Center (FGSC) no. 9519] was used for the study. The high level of nutrients was necessary to ensure the canceling of the (possible) chemotaxis-driven growth directionality. The FM4-64 dye (Invitrogen Ltd.) was used as a marker for Spitzenkörper.

### Time-Lapse Microscopy and Image Analysis.

Live-cell imaging used an inverted laser-scanning microscope (Zeiss Axio Observer Z1 with LSM 5 Exciter RGB, Carl Zeiss) with photomultiplier detectors. Fluorescence and bright-field time-lapse images were captured simultaneously and analyzed using image processing software (Zen 2008, Carl Zeiss).

### Growth Experiments on Agar and Microfluidic Structures.

Control measurements for fungal growth in nonconstraining environments were performed on 1% wt/vol malt extract media using somatic hyphae at the edges of the colony. Hyphal growth rates were measured by tracking the position of the extreme hyphal apices in subsequent frames. Fungal growth was recorded for the period needed to observe hyphal behavior from the entry in, to the exit from, the microfluidic network of interest, which require ∼20 min for a straight 100-µm channel. Due to the more convoluted geometries and the presence of multiple hyphae, in many instances, the image recording lasted more than 1 h. To measure the rates of microtubule polymerization within the apical compartment and to distinguish this from motility, the positions of individual filament ends were tracked frame-by-frame, following a methodology reported previously (25).

### Statistical Analysis.

Statistica 7.1 (Statsoft Inc.) and GraphPad Prism 6.01 (GraphPad Software Inc.) were used for statistical analysis and correlation tests. Statistical analyses included calculating the mean and SD values of parameters measured: i.e., position, alignment with the hyphal axis, polymerization rate for microtubules, times before reappearance of the Spitzenkörper, and hyphal bulge dimensions, over the total number *n* data points, reported for each instance. Statistical analyses included all accumulated data from at least 20 separate experiments (unless otherwise stated).

A full account of the methods is presented in *SI Appendix*.

## Supplementary Material

Supplementary File

Supplementary File

Supplementary File

Supplementary File

Supplementary File

Supplementary File

Supplementary File

Supplementary File

Supplementary File

Supplementary File

Supplementary File

Supplementary File

Supplementary File

Supplementary File

Supplementary File

Supplementary File
