# Supplementary material for: Intracellular mechanisms of fungal space searching in microenvironments
Source: Proc Natl Acad Sci U S A. 2019 Jun 18;116(27):13543–52. doi: 10.1073/pnas.1816423116 (PMC6613077; doi:10.1073/pnas.1816423116)
Supplement: Supplementary File [file pnas.1816423116.sapp.pdf]

SUPPLEMENTARY INFORMATION

Intracellular mechanisms of fungal space searching in microenvironments

Marie Held, Ondrej Kaspar, Clive Edwards, Dan V. Nicolau

1. Supplementary text and figures

1.1. Fungal growth on flat agar surfaces, and in closed non-constraining PDMS geometries

1.1.1. Experimental system

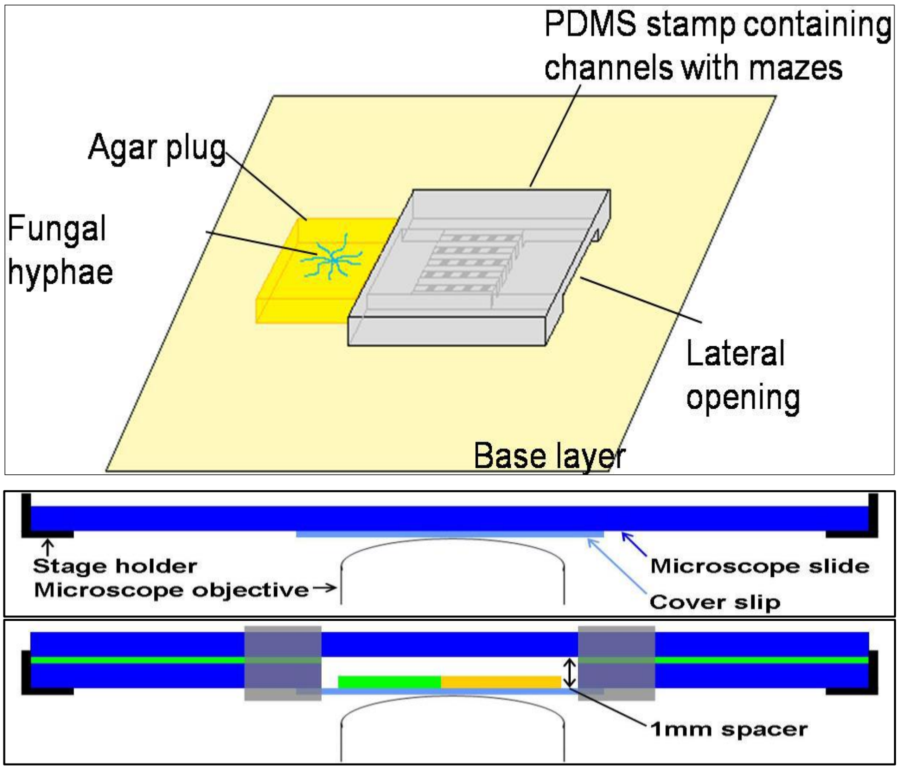

Figure S1. Top panel: top view of the overall experimental setup (optical imaging system not shown). Bottom panel: side view of the experimental implementation for fluorescence studies (not to scale). Top: Normal implementation requirements. Bottom: Experimental assembly used in this study.

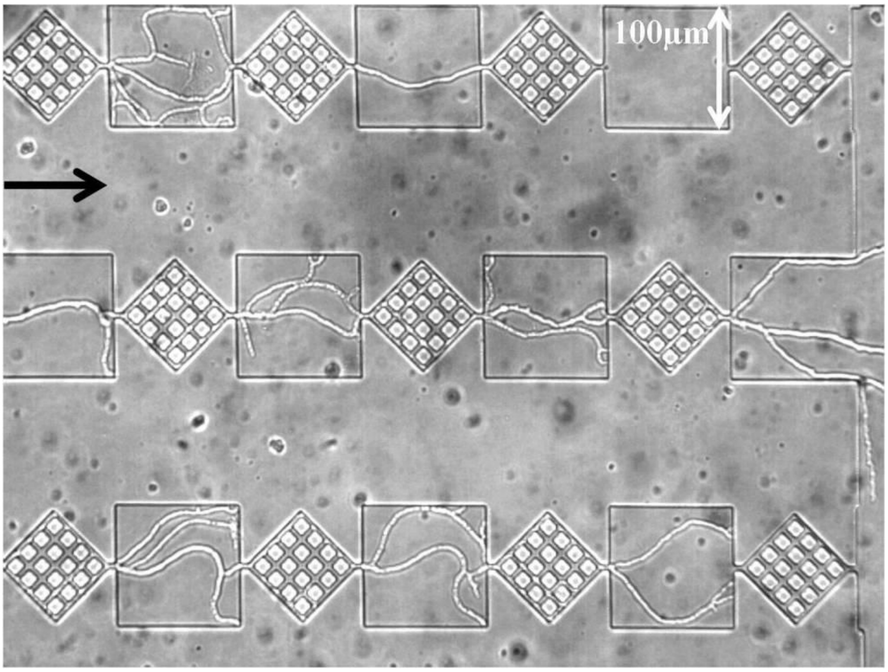

Figure S2. *Neurospora crassa* exploring in parallel a series of 100 µm-wide chambers. The black arrow indicates the direction the exploration of the set of networks. The overall trajectories of the exploring hyphae present with a trend from left to right, which is induced by the layout of the individual microfluidic networks and connecting chambers.

www.pnas.org/cgi/doi/10.1073/pnas.1816423116

1.1.2. Calculations for the design and operation of microfluidics chambers

Oxygen diffusivity. The diffusion coefficient of molecular oxygen in water can be estimated using the Stokes-Einstein equation. Assuming that water and oxygen molecules are ideally spherical and that the channel is filled arbitrarily slowly, the diffusion coefficient D of oxygen in water is

$$D = \frac{k_B T}{6\pi\eta R_0} \qquad \text{Eq. 1}$$

where k_B is the Boltzmann constant, T is the absolute temperature, η is the dynamic viscosity of the solvent, and R_0 is the hydrodynamic radius of the diffusing particles. R_0 is the effective radius of the particle that experiences resistance from the viscous solution:

$$R_{0-Oxygen} = 1.21 \cdot 10^{-10} m \qquad \text{Eq. 2}$$

The temperature T = 296.15 K used in the calculation corresponds to the average room temperature of 21°C measured over the duration of the experiments. The dynamic (or absolute) viscosity of water depends on T according to Arrhenius-Andrade equation

$$\eta = \eta_0 \cdot e^{\frac{E_A}{RT}} \qquad \text{Eq. 3}$$

where η_0 is the material viscosity under standard conditions, E_A is an activation (or transposition) energy, and R is the absolute gas constant. Adams et al. (1) measured the T-dependence over the range 274 K < T < 373 K, yielding a simplified empirical formulation

$$\eta = A \cdot 10^{\frac{B}{T-C}} \qquad \text{Eq. 4}$$

with empirical constants $A = 2.414 \cdot 10^{-5}\ kg \cdot m^{-1} \cdot s^{-1}$, B = 247.8 K, and C = 140.0 K. Therefore, the viscosity of water at average room temperature during the experiments (23°C) is

$$\eta_{296.15} = 9.33 \cdot 10^{-4} Pa \cdot s \qquad \text{Eq. 5}$$

Using this viscosity, the diffusion constant of an oxygen molecule in aqueous solution is

$$D = 1.92 \cdot 10^{-9} \frac{m^2}{s} \qquad \text{Eq. 6}$$

This value is less than $3.4 \cdot 10^{-9}\ m^2/s$, reported in PDMS (2), which is permeable to both oxygen and carbon dioxide.

Diffusivity of nutrients. By modelling maltose and oxygen molecules as ideally spherical molecules, and assuming that the channel is filled arbitrarily slowly, the diffusion of oxygen in water can be described using the Stokes-Einstein equation. Substituting a hydrodynamic radius for maltose of $R_{0\text{-Maltose}}$ = 5 Å (3) in Eq. 1., and assuming the previously quoted values for the other constants, yields the diffusion constant

$$D_{Maltose} = 4.65 \cdot 10^{-10} \frac{m^2}{s}$$

This value is approximately four times smaller than that for oxygen (~ $2 \times 10^{-9}\ m^2/s$). The time needed for the maltose to diffuse a distance *x* ~ 100 µm (the length between the test structure, e.g., diamond, and larger volumes of nutrients, e.g., opening or 100 µm x 100 µm 'plazas') can thus be calculated as

$$t = \frac{\langle x^2 \rangle}{2 \cdot D_{Maltose}} = \frac{100^2 \cdot 10^{-12}\, m^2}{2 \cdot 4.651 \cdot 10^{-10} \frac{m^2}{s}} = 10.75\, s$$

This time is three orders of magnitude shorter than the time taken by a typical apical extension of *Neurospora crassa* to traverse the test structure, assuming an average extension velocity of ~ 0.006 µm/s. In conclusion, the microfluidics structures provide rapid and high level of nutrients, thus not impacting on fungal growth.

Table S1. Relevant parameters for the operation of fungal confinement chips

Medium	Diffusivity of O_2 (m^2/s)	Diffusivity of sugars (m^2/s)
**Air**	$1.8 \cdot 10^{-5}$	-
**Water**	$1.9 \cdot 10^{-9}$	$4.7 \cdot 10^{-10}$ for maltose, cf. above
**Agar**	$2.4 \cdot 10^{-9}$, 2% agar (4)	$2.9 \cdot 10^{-10}$ for sucrose (5)
**PDMS**	$3.4 \cdot 10^{-9}$ (2)	-

1.1.3. Penetration depth of *Neurospora crassa* hyphae into an agar plate

The fungal growth on flat agar surfaces benefits from unencumbered access to oxygen. In contrast, the oxygen needs to diffuse through PDMS to the hyphae growing in closed, but non-constraining environments, or through the aqueous solutions in the microfluidics channels. It appears however that the lower oxygen content in closed but non-confining PDMS structures, compared with the open agar surfaces, does not affect hyphal growth. Indeed, the following images show vertical cross-sections of an agar plate, onto which a fungal colony has been inoculated. Fungal growth then occurs on, or at a shallow depth below the surface, but there is also some deeper penetration, beyond the accessible range of a confocal microscope.

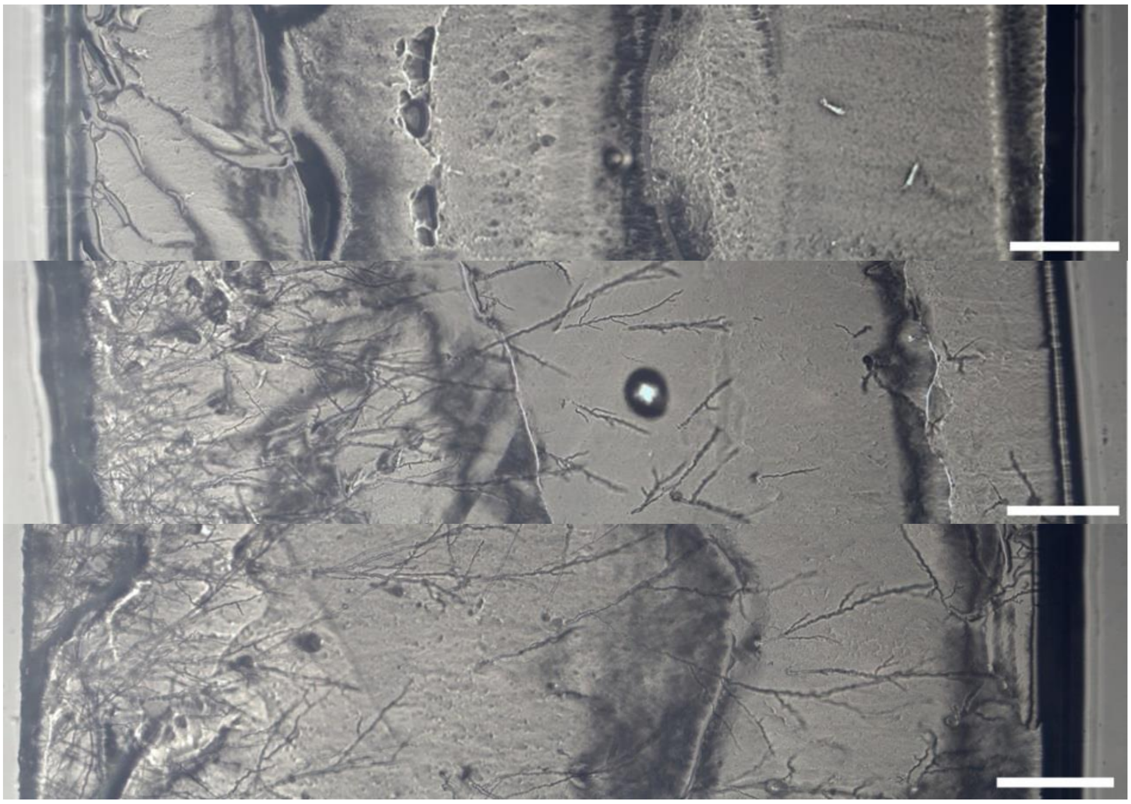

Figure S3. Micrographs of the hyphal vertical penetration in agar material. Sections away from the edge of the agar plug: top: at the edge (penetration depth of the bulk of hyphae ~80 μm); middle: 0.5 cm from the edge (penetration depth ~670 μm); and bottom: 1 cm from the edge (penetration depth ~approximately 2260 μm). In the middle of the agar plate, the fungus penetrates the entire depth of the agar plate, i.e., more than 2 mm. The scale bar = 500 μm.

1.1.4. Equivalence between conditions of growth on agar and in control PDMS chambers

The environments presented to hyphae by agar surfaces and PDMS structures are not geometrically similar, raising the possibility of differences in fungal growth. First, the hyphae growing on agar are not physically constrained, as they could even penetrate it for more than 2 mm. However, the PDMS structures used to study the intracellular mechanisms in quasi-

open conditions consist of chambers with widths of 100 µm and heights of 10 µm, which exceeds the diameters of a typical *Neurospora crassa* hypha, i.e., 4-7 µm, consequently excluding physical constraining. Second, on agar the hyphae are directly exposed to air, but in experiments in closed/non-confining environments, the oxygen reaches the hyphae through diffusion through microns-to-millimetres thick PDMS material. Although the diffusivity of oxygen in air ($1.8 \cdot 10^{-5}$ m^2 s^{-1}) is approximately four order of magnitude higher than in PDMS ($3.4 \cdot 10^{-9}$ m^2 s^{-1} (6)), the capacity of hyphae to penetrate agar, which has a lower diffusivity of oxygen ($2.4 \cdot 10^{-9}$ m^2 s^{-1}) than PDMS, suggests that oxygen is present at levels not impacting fungal growth. Third, the diffusivity of sugars in water (the carrier of nutrients for in closed/non-confining PDMS structures) is estimated at $4.7 \cdot 10^{-9}$ m^2 s^{-1}, which is more than an order of magnitude higher than for agar, i.e., $2.9 \cdot 10^{-10}$ m^2 s^{-1}, which suggests that nutrient levels will not negatively affect fungal growth.

1.1.5. Intracellular mechanisms for *Neurospora crassa* growth on external and internal control

Hyphal growth behaviour and related intracellular processes in PDMS non-constraining environments are similar with those observed on agar (Supplementary Figure S2). First, the cross-sectional apical profiles of Neurospora crassa hyphae are parabolic and symmetrical (Figure 2a for internal control; Supplementary Figure S4 for external control). Second, the Spitzenkörper is centred at the hyphal apex (Supplementary Movie S1, and Supplementary Figure S5), with small periodic oscillations orthogonal to the direction of growth (Supplementary Movie S2). Third, the microtubules are predominantly orientated parallel to the longitudinal hyphal axis (Figure 2a for internal control; Supplementary Figures S4 and S6 for external control). For instance, in the apical regions, a majority of microtubules (53%) deviate by less than 10° from the polarisation axis, and 84% deviate by less than 20°, with an overall mean deviation angle of 11.7° ± 9.5° (n = 453 microtubules measured in 20 hyphae, Figure 2b, Supplementary Movie S3). By contrast, in subapical compartments the angular deviations of microtubules are larger, i.e., 21% microtubules presenting a deviation of less than 10°, and 46% less than 20°, with an overall mean deviation angle of 26.8 ± 20.1° (n = 852 microtubules measured in 20 hyphae; Figure 2b and Supplementary Movie S4).

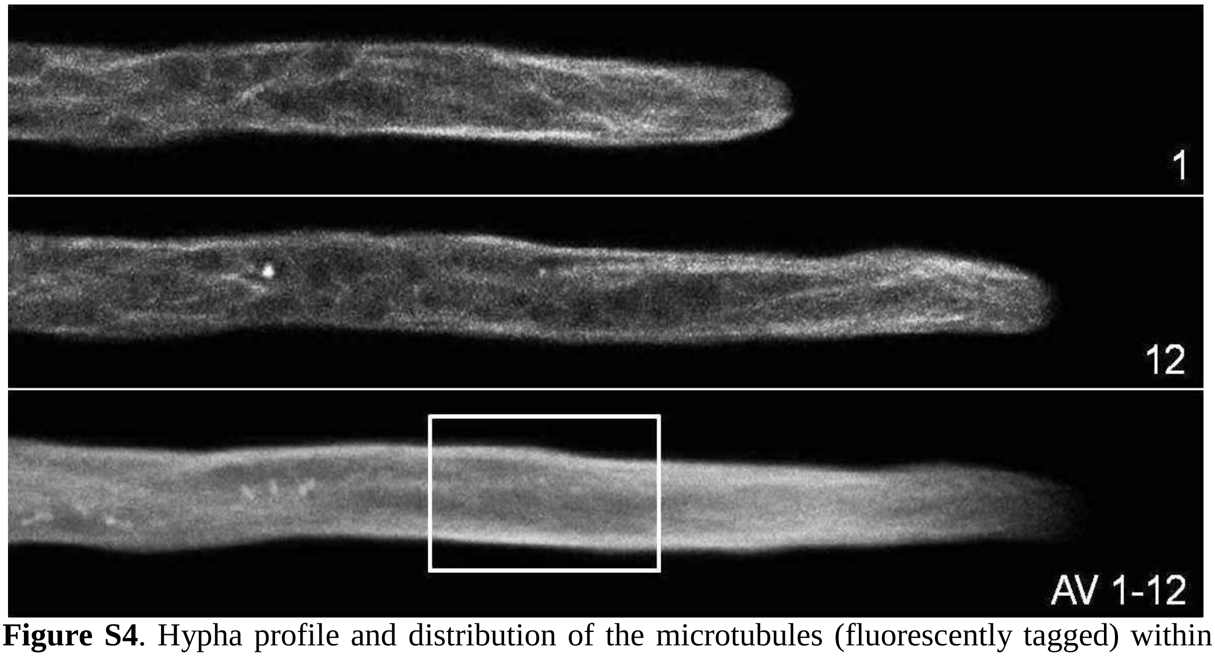

Figure S4. Hypha profile and distribution of the microtubules (fluorescently tagged) within growing hypha. Top: First and last frame and overlay of an image series of the fluorescence signal of *Neurospora crassa* GFP on agar. The spatial distribution of the microtubules (presented further in Figure S7) is quantified in the indicated rectangle.

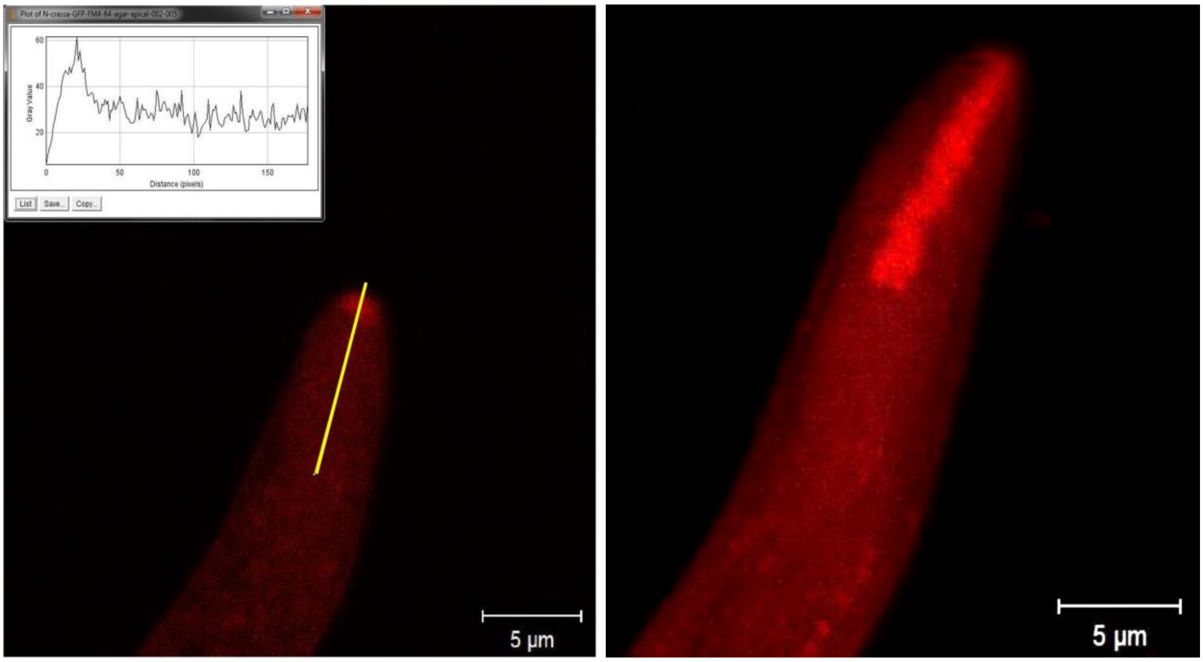

Figure S5. FM4-64 intensity profile and Spitzenkörper trajectory in the apical compartment of *Neurospora crassa* GFP on agar. Left: Apical compartment of a *Neurospora crassa* GFP hypha loaded with FM4-64 and grown on agar. The yellow line indicates the line along which the intensity profile (inset) was recorded. The abscissa correlates to the line starting at the top end, which lies outside the hypha. Following the profile, the intensity spikes at the location of the Spitzenkörper and then decreases rapidly to the constant moderate intensity level of the hyphal cytoplasm. Right: Overlay of the maximum intensities of 23 images of a time series showing the Spitzenkörper in the apical compartment. Images were recorded at intervals of 3.15 s. The overlay resulted in a pronounced trajectory of the Spitzenkörper. The intensity decrease of the trajectory was caused by photobleaching.

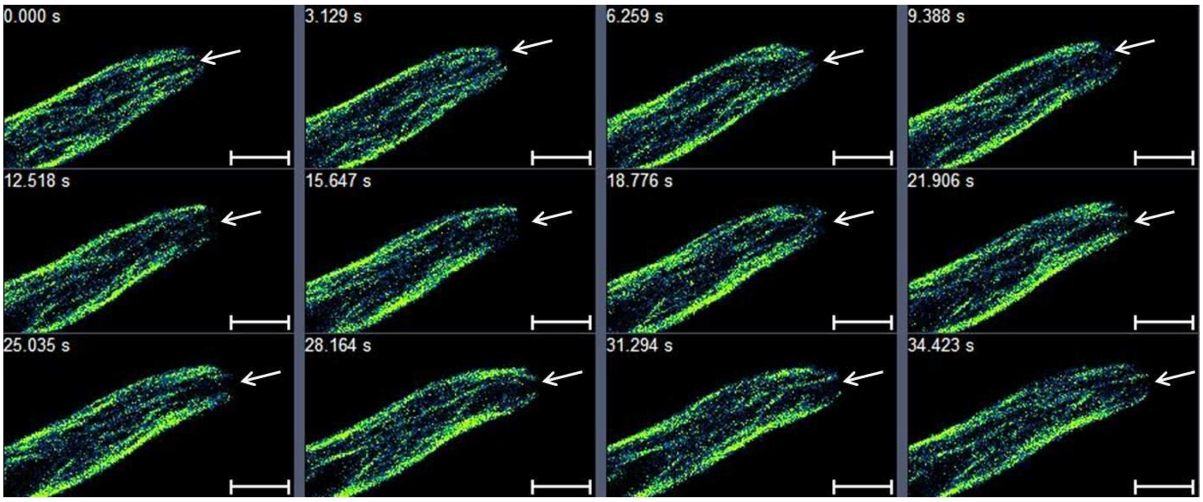

Figure S6. Time series of the microtubule distribution in a *Neuropsora crassa* GFP hypha growing on plain agar. The images were recorded in intervals of 3:1 s. The white arrows mark the 'voids' of the microtubule distributions near the apical cell wall correlating with the position of the Spitzenkörper (identified in simultaneously recorded bright field images). Scale bar: 5 µm

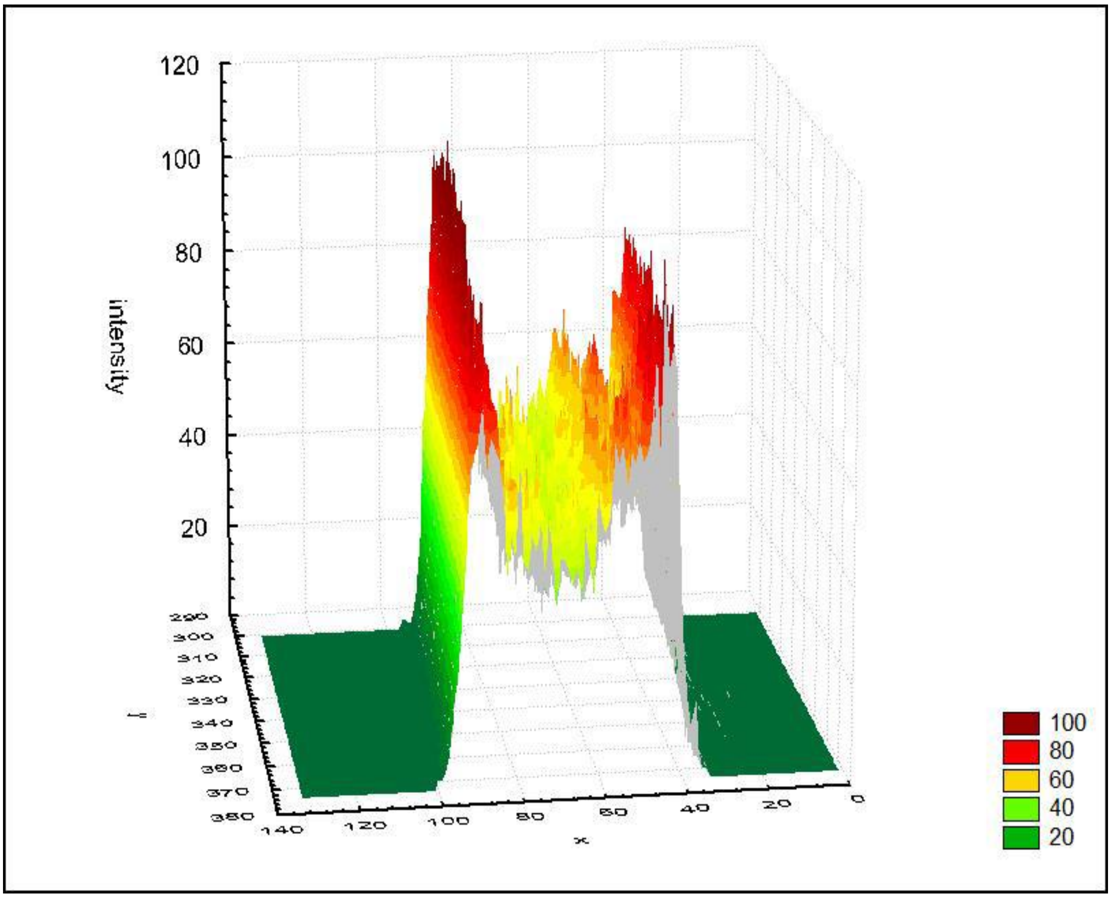

Figure S7. 3D representation of the average intensity values due to microtubules in the marked box in the overlay image in Figure S4. Both representations show that the microtubule distribution across the cross section of the hypha is inhomogeneous characterised by an accumulation of filaments close to the hyphal cell wall.

The following histograms present the spatial distributions of the microtubule relative to the hyphal polarisation axis, in the apical and sub-apical compartments; and in agar and microfluidic environments.

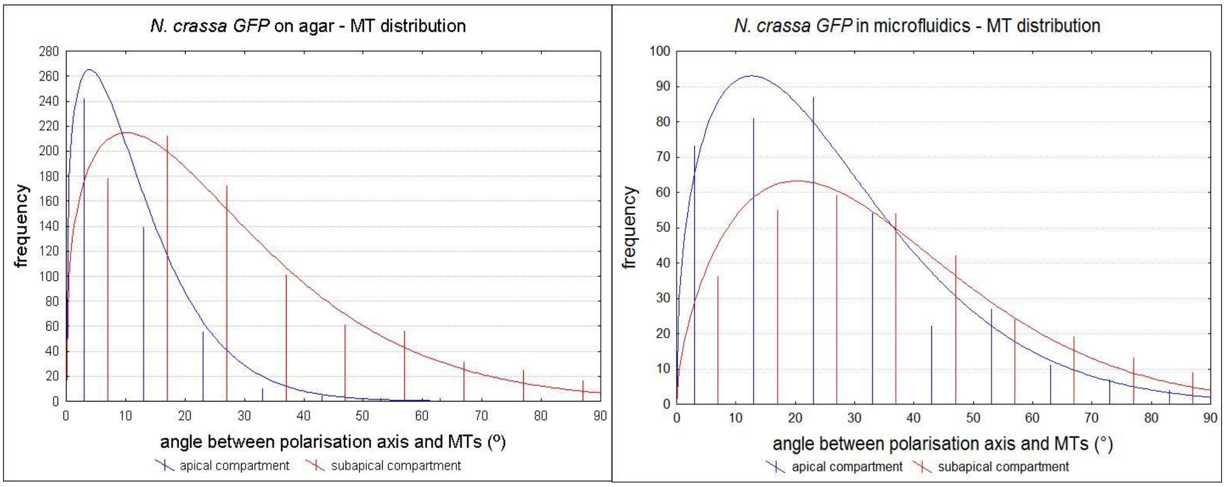

Figure S8. Statistical distributions of microtubules for hyphae growing on/in agar, and on PDMS microfluidics, respectively, in the apical, and subapical regions of the hyphae.

Table S2. Microtubule orientation on agar and in confined spaces

Interval	Agar		Microfluidics	
	Apical	Subapical	Apical	Subapical
0° to 10°	53%	21%	20%	12%
0° to 20°	84%	46%	69%	29%
0° to 80°	100%	98%	99%	97%
45° to 90°	1%	18%	16%	26%

This statistical analysis of the orientation of the microtubules shows that, aside from minor shifts of alignment versus the hyphal axis in closed/non-constraining environments compared with hyphae on agar (from ~5° to 12° in the apical compartment; and from 10° to 20° for subapical compartment), the distribution of microtubules has a clear propensity towards hyphal walls in both experimental conditions.

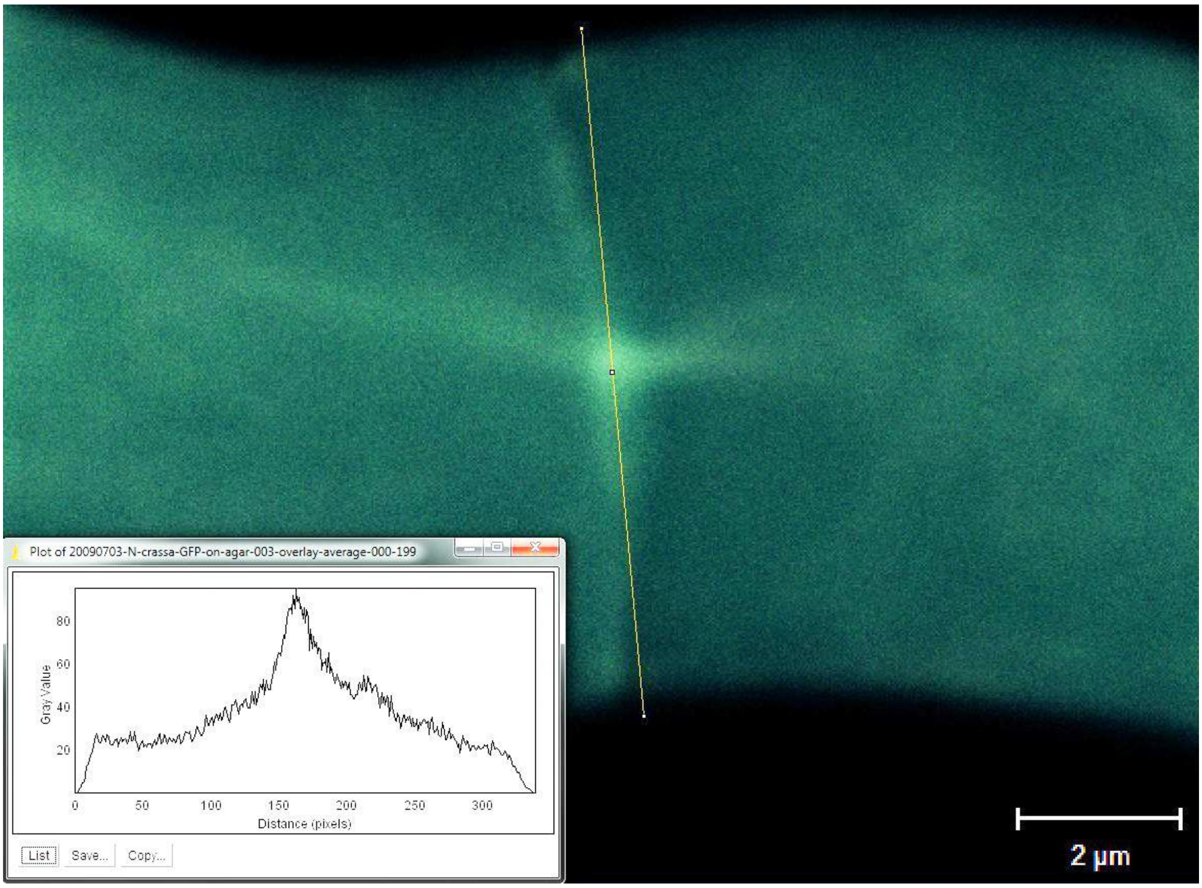

Figure S9. Microtubules passing through a septum placed centrally in the hypha (average over 200 images). The graph in the inset represents the profile of the intensity values along the indicated yellow line.

1.1.6. Intracellular mechanisms of branching for external and internal controls

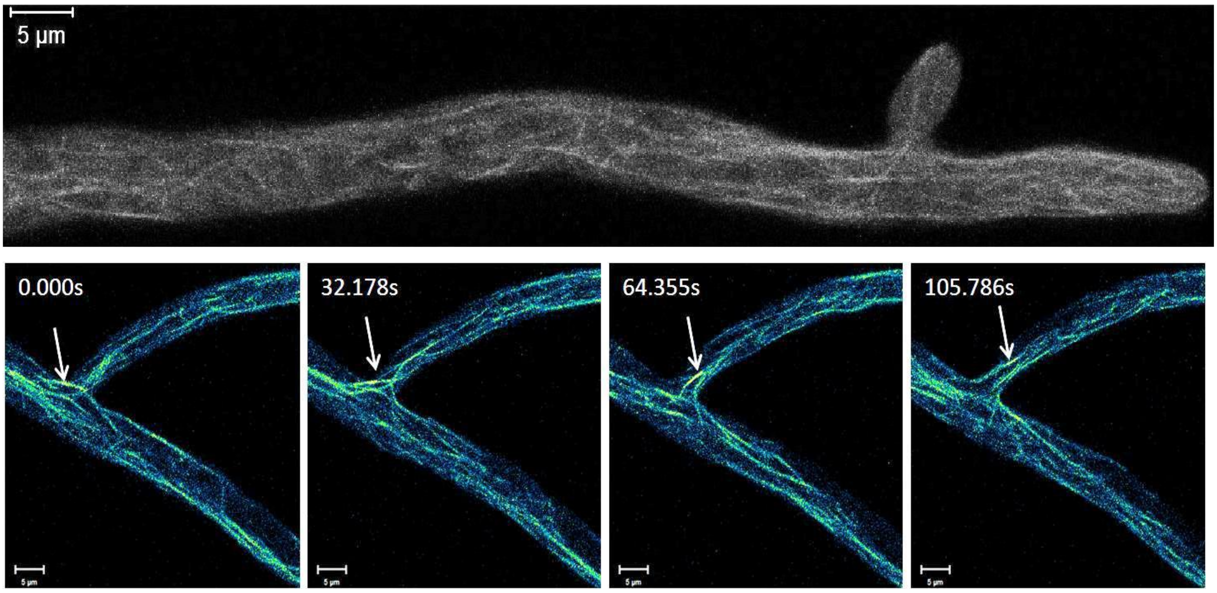

Figure S10. Top: Branching at 45° in *Neurospora crassa* GFP on agar. Bottom: Time series of the microtubule distribution at an established branching point in a *Neuropsora crassa* GFP hypha growing on agar. The white arrows mark the position of a microtubule moving from the parent hypha into the daughter hypha. Scale bar: 5 µm.

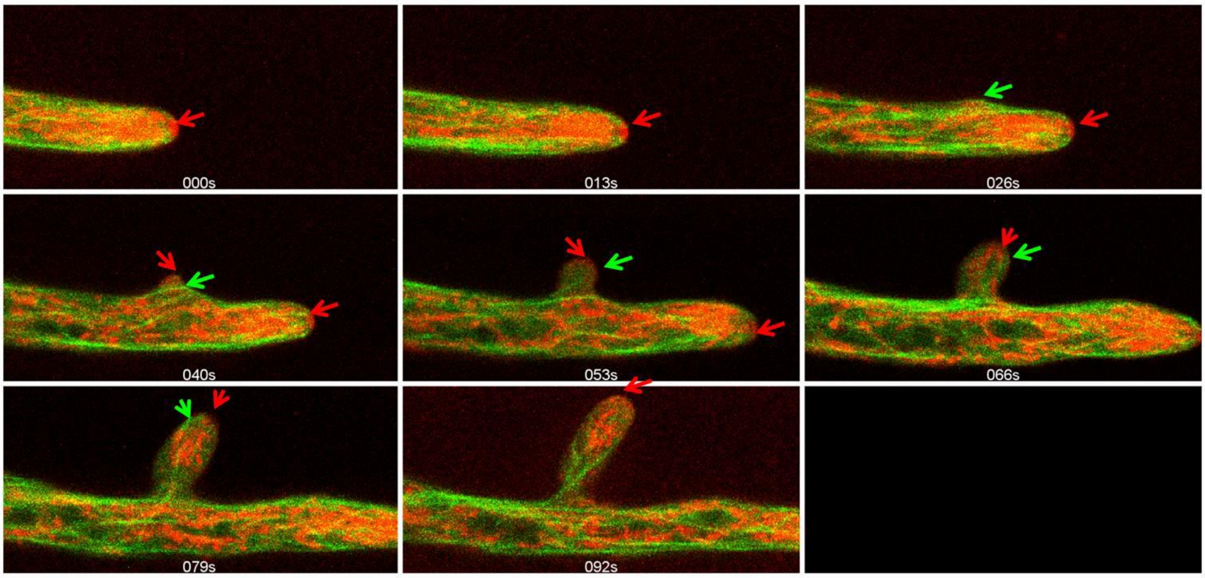

Figure S11. Formation of a lateral branch in non-constraining PDMS structures. Overlay of the fluorescence signal of the microtubules and Spitzenkörper during a branching event. The red arrows indicate the Spitzenkörper and the green arrows indicate the microtubules terminating at the point of hyphal formation (26s) and that extend from the parent into the forming branch (40s - 90s). The hyphal diameter is 4.9 µm.

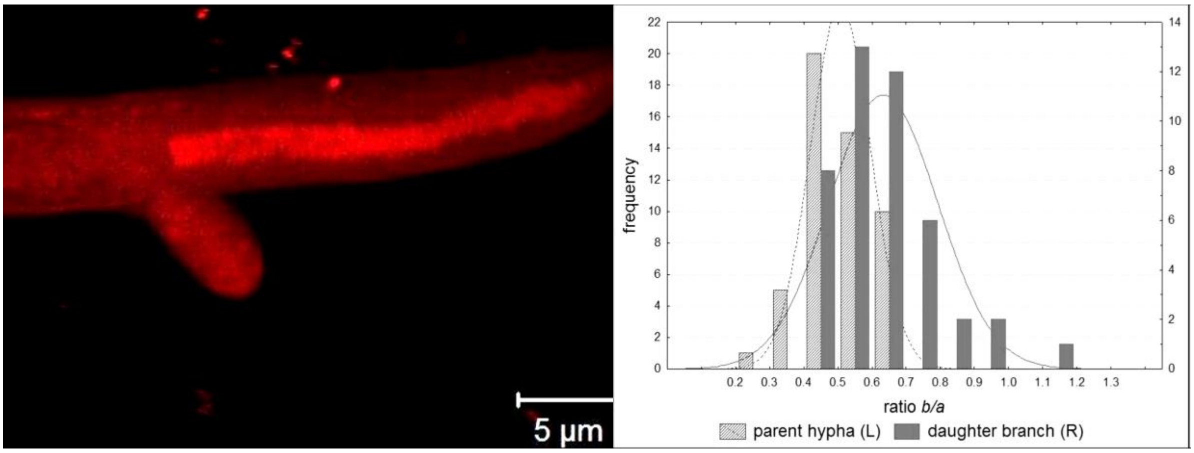

Figure S12. Branching event in a *Neurospora crassa* hypha on agar. Left: Overlay of the maximum intensities of 68 images of Spitzenkörper movement during branching (images were recorded at 5.76s intervals). Spitzenkörper trajectory in the daughter hypha is less clear than that for the parental hyphae due to its movement out of the focal plane, e.g., penetrating agar. Right: Distribution of the Spitzenkörper ellipsoid axis ratios in the parent and the daughter branch. The average ratios are 0.50 and 0.62.

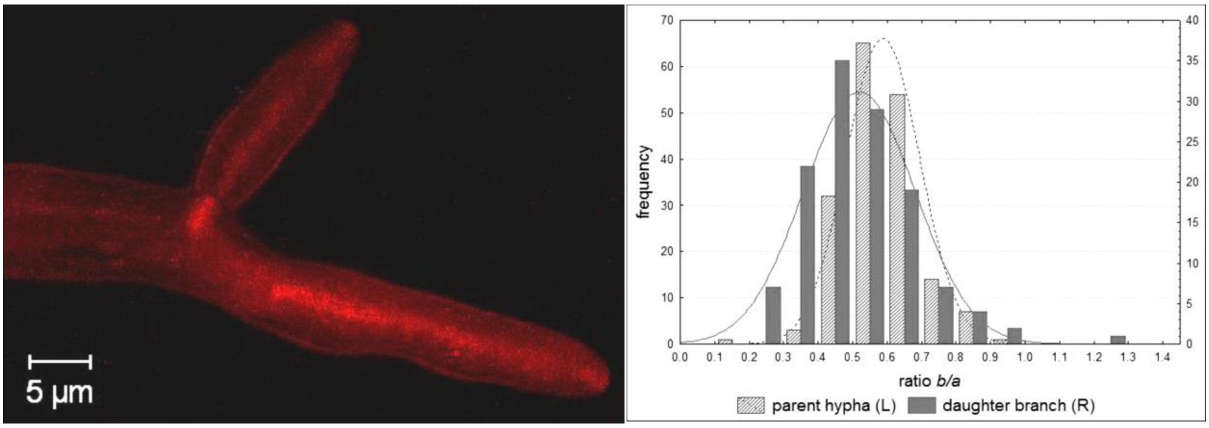

Figure S13. Branching events in FM4-64 loaded *Neurospora crassa* GFP hyphae in wide PDMS channels. Left: Overlay of the maximum intensities of 100 images of a time series depicting the Spitzenkörper motilities during the formation of a branch in a wide PDMS channel. The images were recorded at intervals of 3.9 s. Right: Distribution of the Spitzenkörper ellipsoid axes in the parent and the daughter branch. The average ratios of the short and the long axes are 0.57 for the parent hypha and 0.51 for the daughter hyphae.

1.2. Collision with obstacles at acute angles of approach

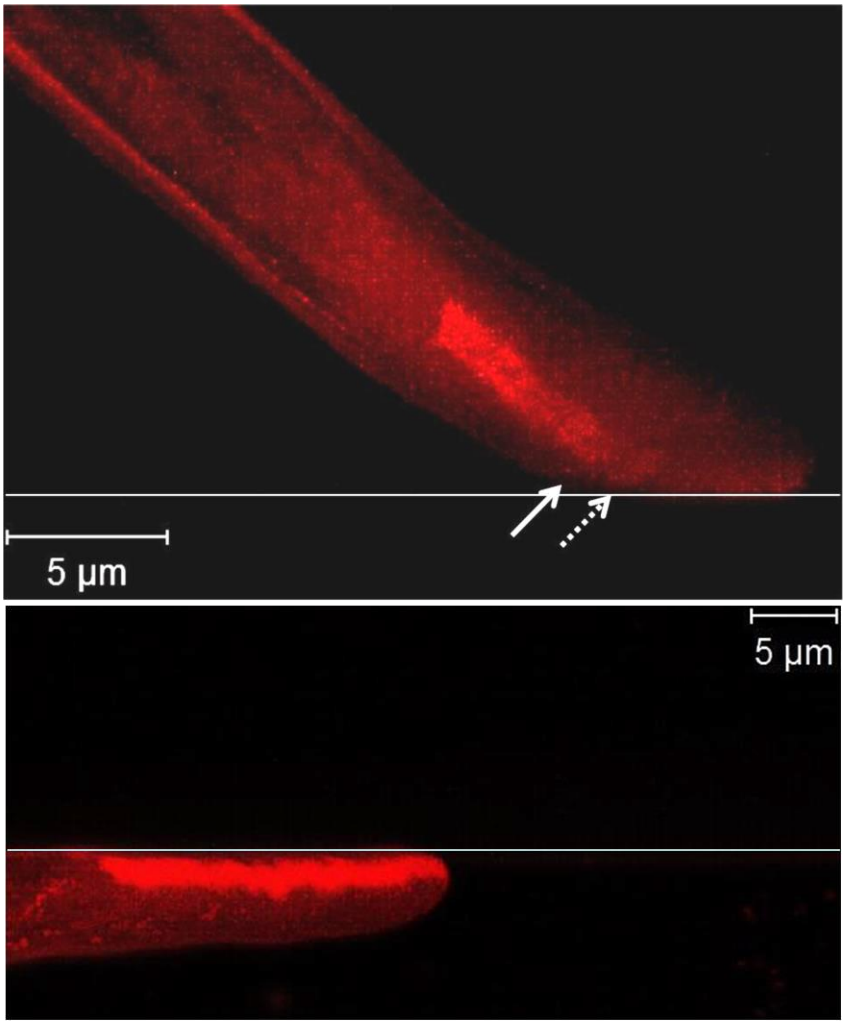

Figure S14. Spitzenkörper trajectories during adaptation and the 'nestling' processes. The solid line in both images indicates the impeding PDMS wall. The solid arrow in the top panel indicates the point where the Spitzenkörper trajectory starts to deviate from the hyphal polarisation axis and the dashed arrow indicates the point where the trajectory aligns parallel to the geometry. Top: Overlay of the maximum intensities of 67 images of a time series depicting the adaption of a hyphal apex to the geometry. The images were recorded at intervals of 4.3 s. The gradual decrease in intensity was caused by photobleaching. Bottom: Overlay of the maximum intensities of 144 images of a time series depicting continuous hyphal 'nestling' to the confining wall. The images were recorded at intervals of 7:4 s. The Spitzenkörper trajectory is permanently dislocated from the central axis of the hypha towards the geometry.

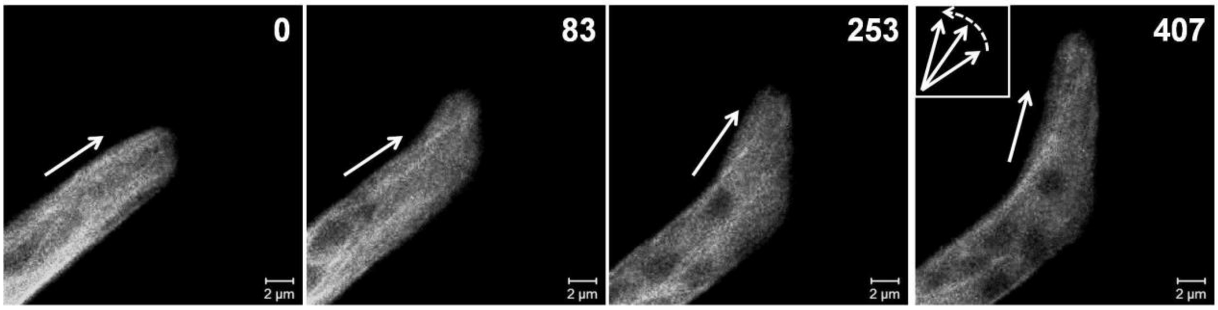

Figure S15. Microtubules in *Neurospora crassa* GFP during 'nestling'. The numbers indicate the elapsed time in seconds. The arrows indicate the direction of the leading microtubules in the apical region. The overlaid arrows in the inset at 407 s represent the change in the direction of the microtubules over the four depicted frames. The reduction in overall intensity was due to photo bleaching effects during the long-term observation.

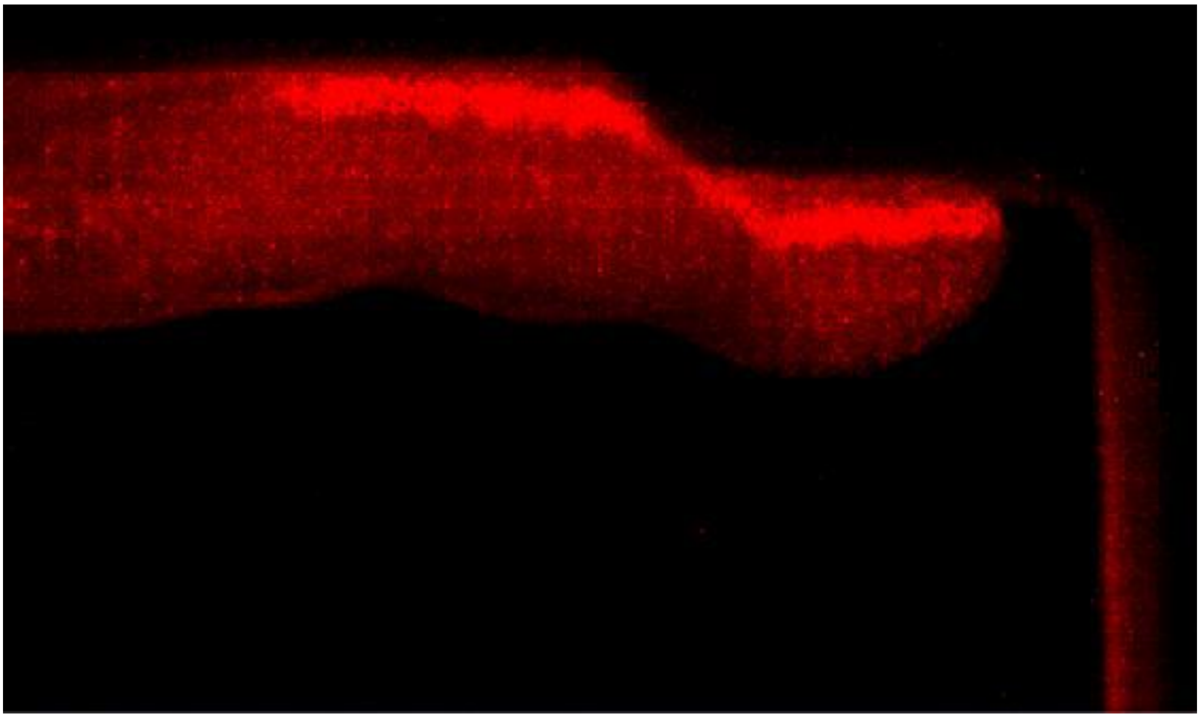

Figure S16. Spitzenkörper motility during ‘nestling’. Overlay of the maximum intensities of 166 images of a time series depicting a successful corner turn. The images were recorded at intervals of 25.4 s. The Spitzenkörper remained located close to the confining geometry in the direction closest to the directional memory.

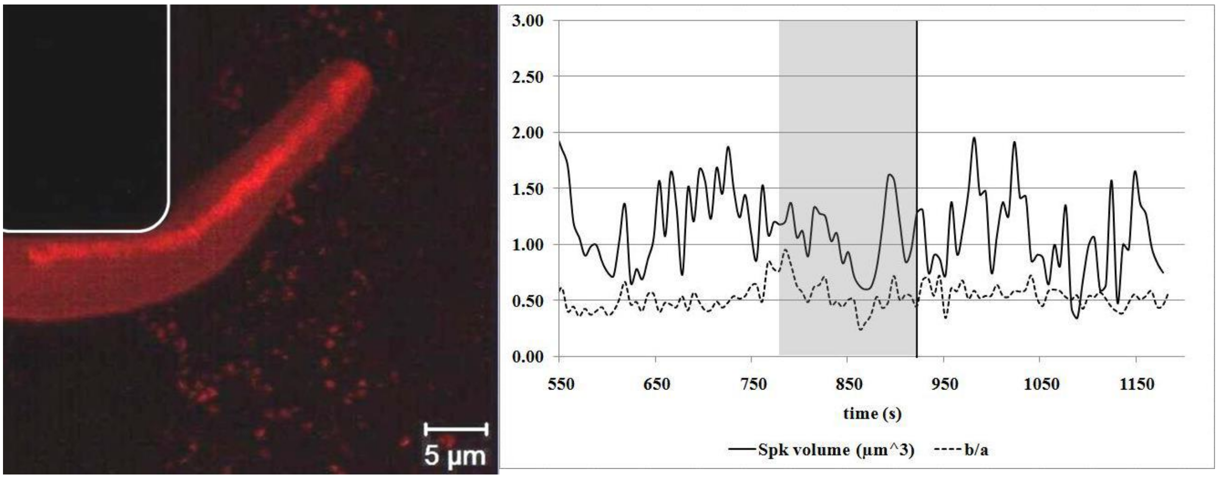

Figure S17. Spitzenkörper trajectory. Left: Overlay of the maximum intensities of 76 images of Spitzenkörper trajectory during the re-adaption to the initial growth direction after deviation by an obstacle (90 corner, white line). The images were recorded at 5.9 s intervals. Right: Time course of the Spitzenkörper volume and the ratio of the ellipsoid axes (b/a) from 550 s to 1200 s in the time series on the left. The grey highlighted area indicates the transition phase from attachment and adaptation to the geometry to the re-adaption of the initial growth direction. The solid black line indicates the time of detachment from the rounded corner.

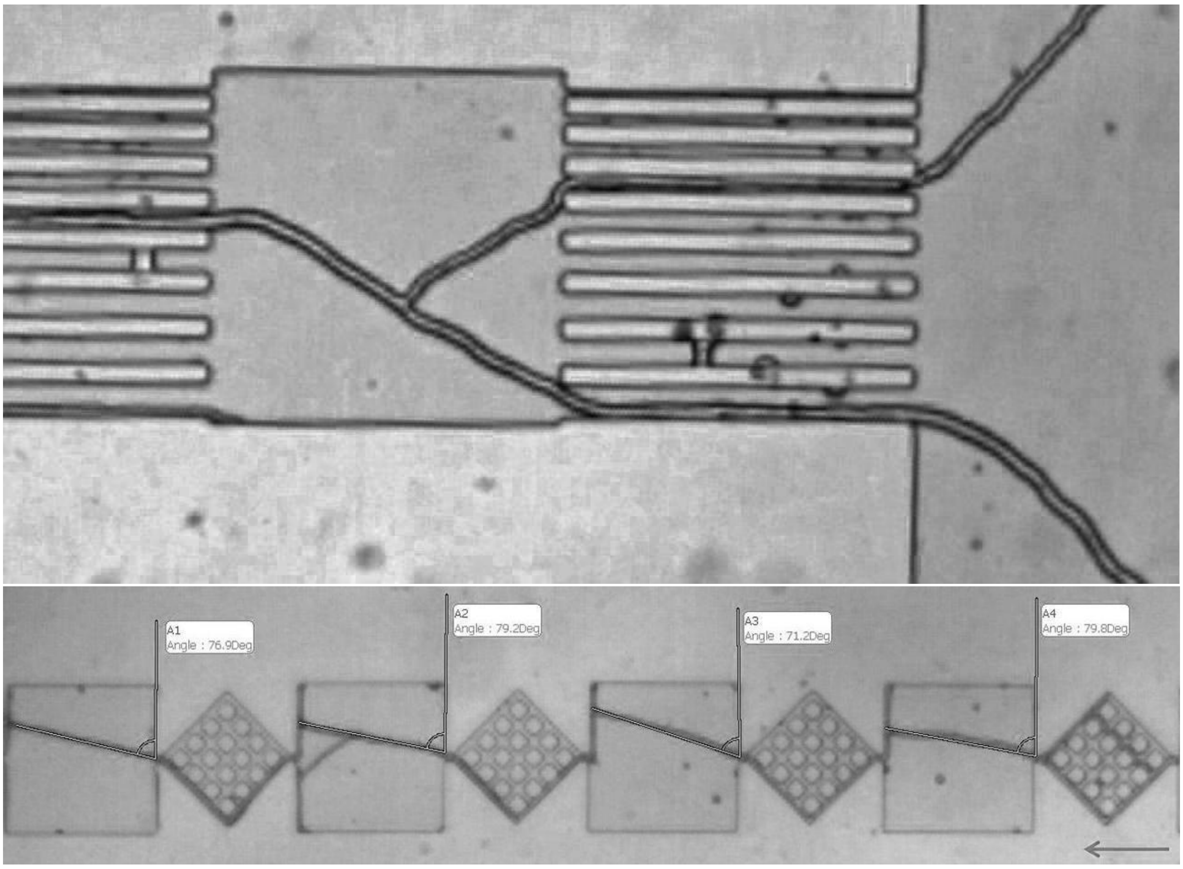

Figure S18. Several instances of the manifestation of directional memory. Top: Both parental (lower side of the image) and the daughter hypha (higher side of the image) preserve their initial direction when the geometry (straight narrow channels) allows it. Bottom: Initial direction of growth is preserved for approximately 800 µm during passage through four diamond and four plaza structures.

1.3. Frontal collision with obstacles

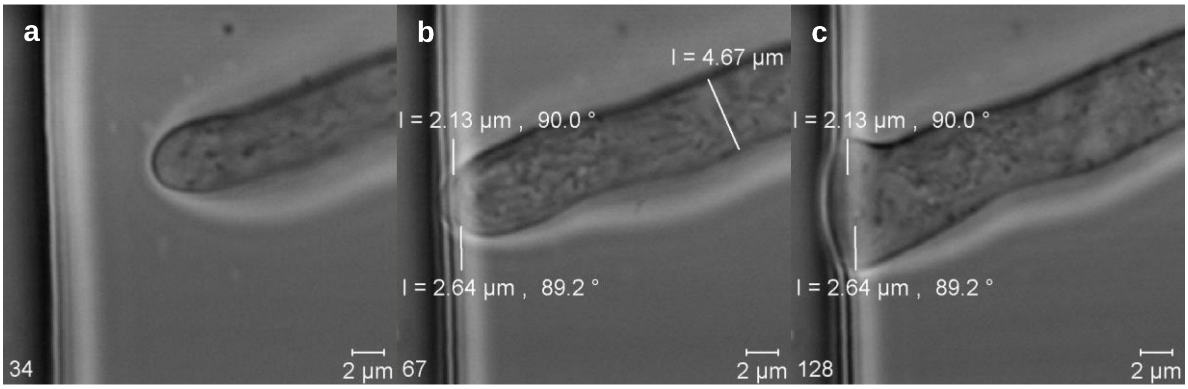

Figure S19. Parameters of the apical split indicated in selected images of a time series of a *Neurospora crassa* GFP hypha colliding head-on with a PDMS wall. The numbers in the bottom left corner indicate the number of the frame in the image series. Left: Frame 34 of an apical split image series. The hypha approaches the PDMS wall on the left head-on. Middle: Frame 67 of an apical split image series. This frame marks the beginning of the collision incident because the hypha is the closest to the wall without any distortions in the apical cell wall. The hyphal diameter is measured in the beginning frame for all collision events. The two distances measured parallel to the wall at the apex indicate the difference of the cell wall position in this beginning and the end frame. Right: Frame 128 of an apical split image series. This frame marks the end of the collision incident because this is the last frame in which the hyphal extension is characterised by uniform bulging. In the succeeding frames, the extension is characterised by the directed extension of the two initiated daughter branches away from the collision point. The two distances measured parallel to the wall at the apex indicate the difference of the cell wall position in this end and the beginning frame of the image series.

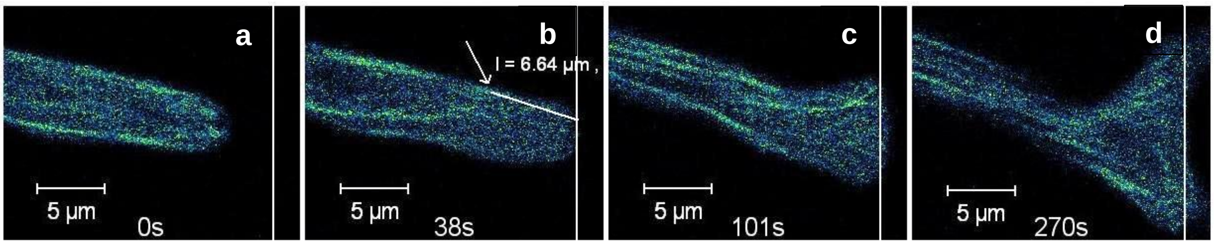

Figure S20. Microtubules in *Neurospora crassa* GFP during ‘hit & split’. The white vertical line indicates the PDMS wall the hypha collided with. At 0 s, the microtubules extended up to the cell wall of the apical dome and were aligned mostly parallel to the growth axis. The second image was recorded 19 s after the collision, which represents the maximum retraction of microtubules of 6.6 µm from the collision point, indicated by the arrow. The frame at 101 s represents the snapshot with the maximum extension of the bulges, which marks the transition to polarised growth of the forming daughter hyphae. The microtubules resumed extension to the apical dome. The frame at 270 s represents an example of the established microtubule distributions in the daughter hyphae.

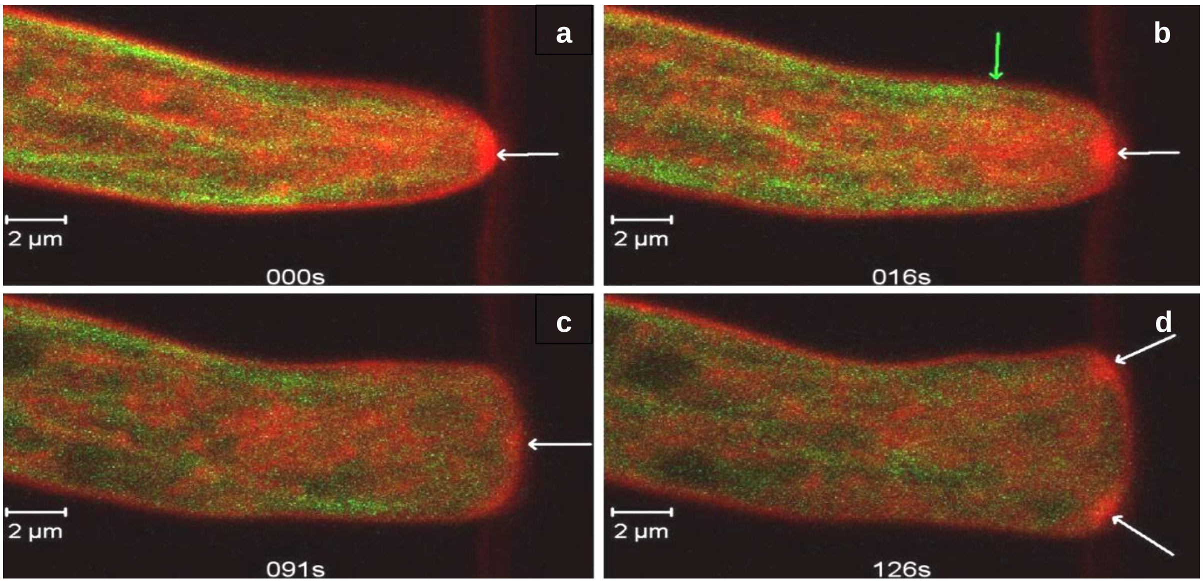

Figure S21. Motility of microtubules and Spitzenkörper during apical hit & split. Four frames of a time series taken during an apical split showing the overlay of the GFP and FM4-64 signals. The selected frames depict the times of collision, maximum distance of the receding microtubules, Spitzenkörper disintegration and establishment of daughter polarisation axes. The white arrows indicate the Spitzenkörper in the individual frames and the green arrow indicates the maximum distance the microtubules receded to. Unfortunately, the GFP signal underwent significant photobleaching and differentiation of features decreased.

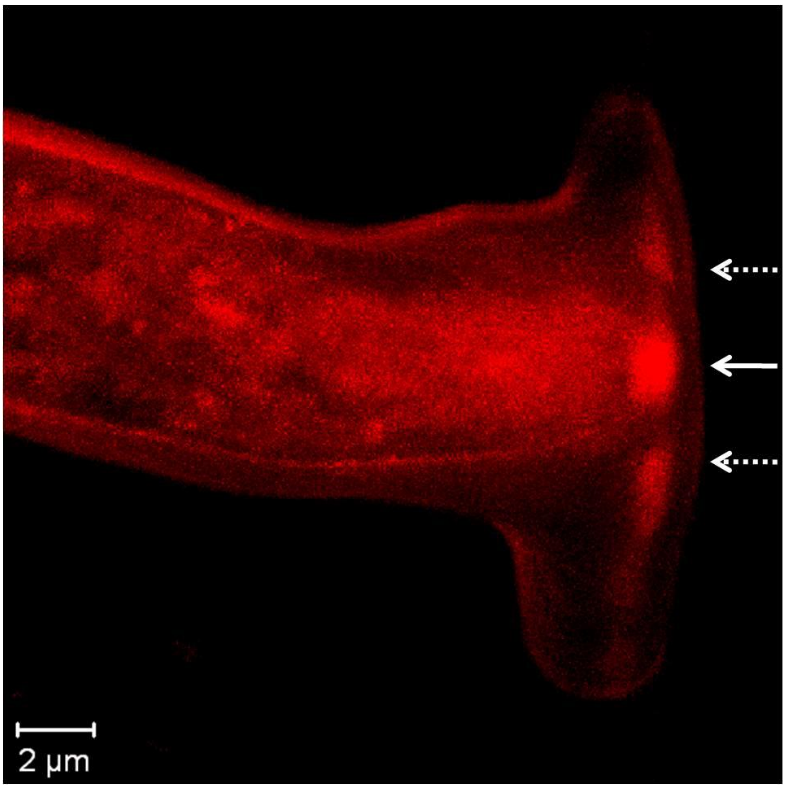

Figure S22. Spitzenkörper trajectories during an apical 'hit & split' *in Neurospora crassa*. Overlay of the maximum intensities of 135 images of a time series depicting the Spitzenkörper motility during collision-induced apical splitting process. The images were recorded at intervals of 4 s. The solid arrows indicate the trajectories of the parent Spitzenkörper and the dashed arrows indicate the trajectories of the daughter Spitzenkörper.

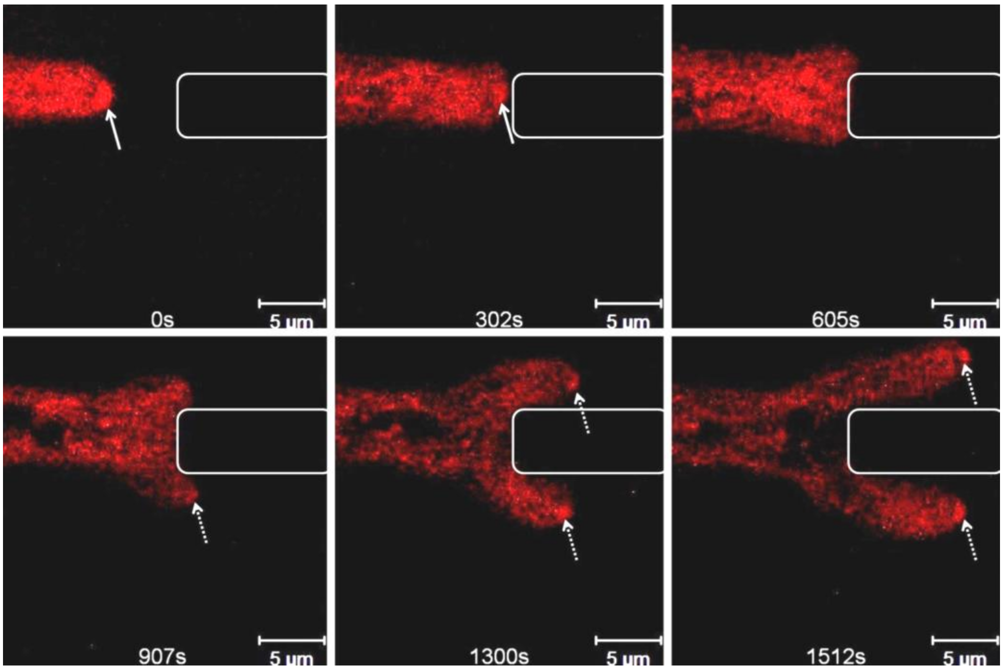

Figure S23. Apical split caused by a head-on collision of a hypha with a PDMS wall dividing individual channels. The solid arrows indicate the parent Spitzenkörper and the dashed arrows indicate the daughter Spitzenkörper.

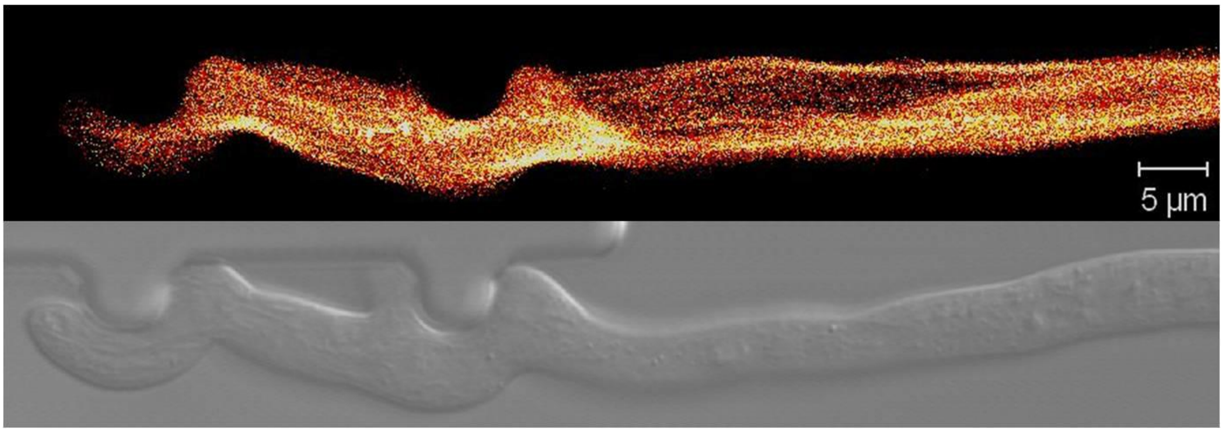

Figure S24. Top: Microtubules in *Neurospora crassa* GFP passing a string of lateral obstacles. Average calculated from 63 images recorded at 11:6 s intervals. Bottom: final snapshot. The microtubule distribution is asymmetric with peaks along the shortest path.

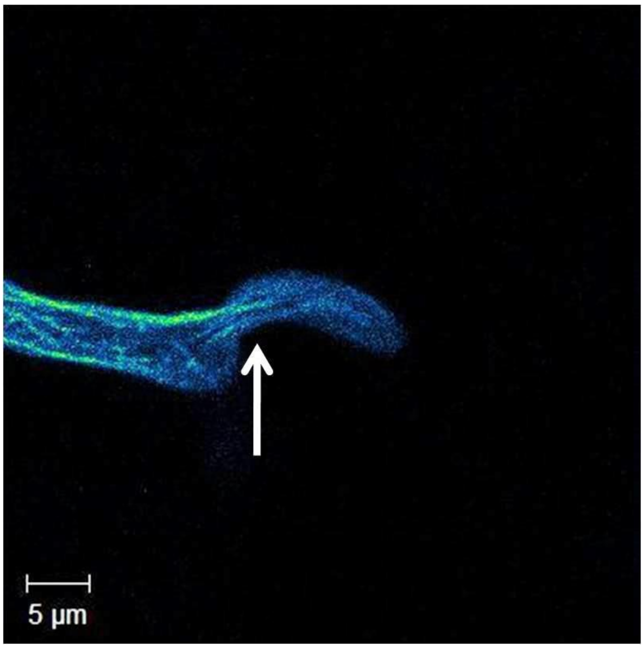

Figure S25. Detail of the distribution of microtubules distribution and intensity drop in the apical compartment of *Neurospora crassa* GFP when a hypha collided with an obstacle at near-orthogonal angle, but able to circumvent it, similarly with the above Fig. S24. The contact with the obstacle is indicated by the white arrow.

1.4. Growth and branching in tightly constraining geometries

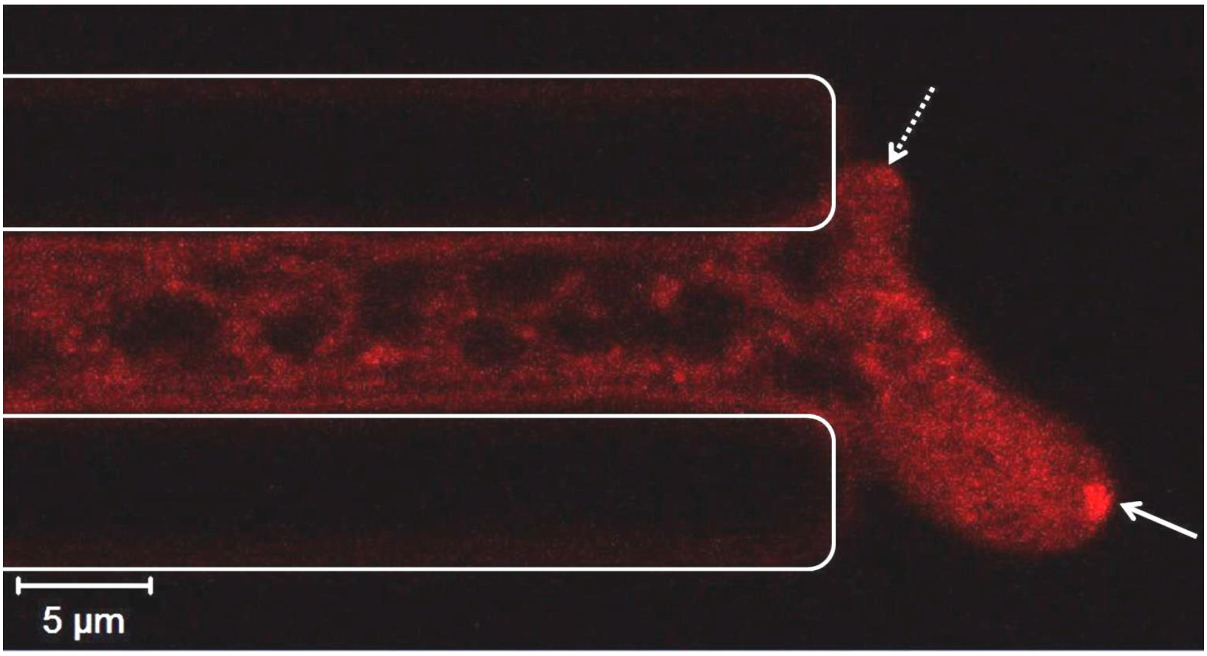

Figure S26. Spitzenkörper mobility and location during branching into channel openings and after bottlenecks. Single focal plane image of a hypha branching after exiting a 5 µm wide channel. The location of the confining PDMS walls is indicated by white outlines.

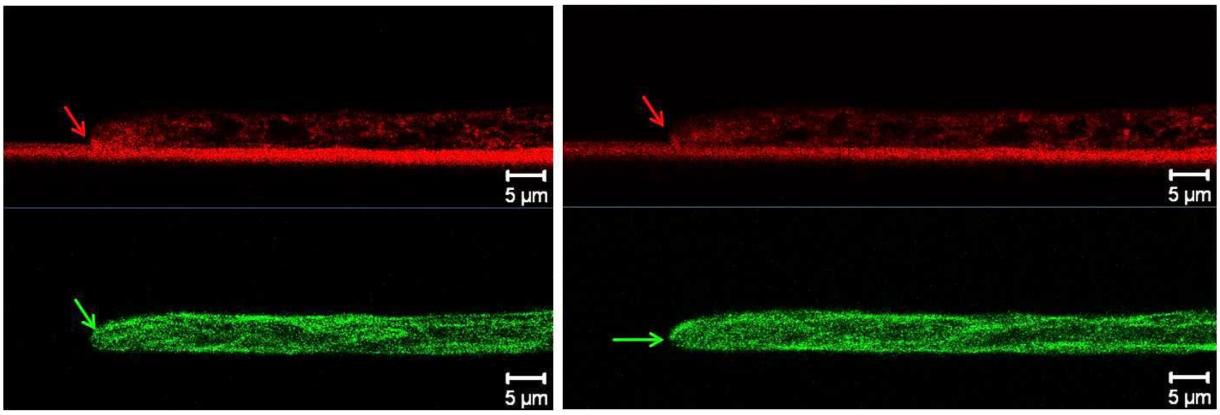

Figure S27. Microtubule distribution (green) and Spitzenkörper (red) locations during 'nestling' in tightly-constraining channels. The hyphae entered the channel from the "North", thus pressing on the "South" edge of the channels. The red arrows indicate the Spitzenkörper in both frames. The green arrows indicate the foremost microtubules in the apical dome.

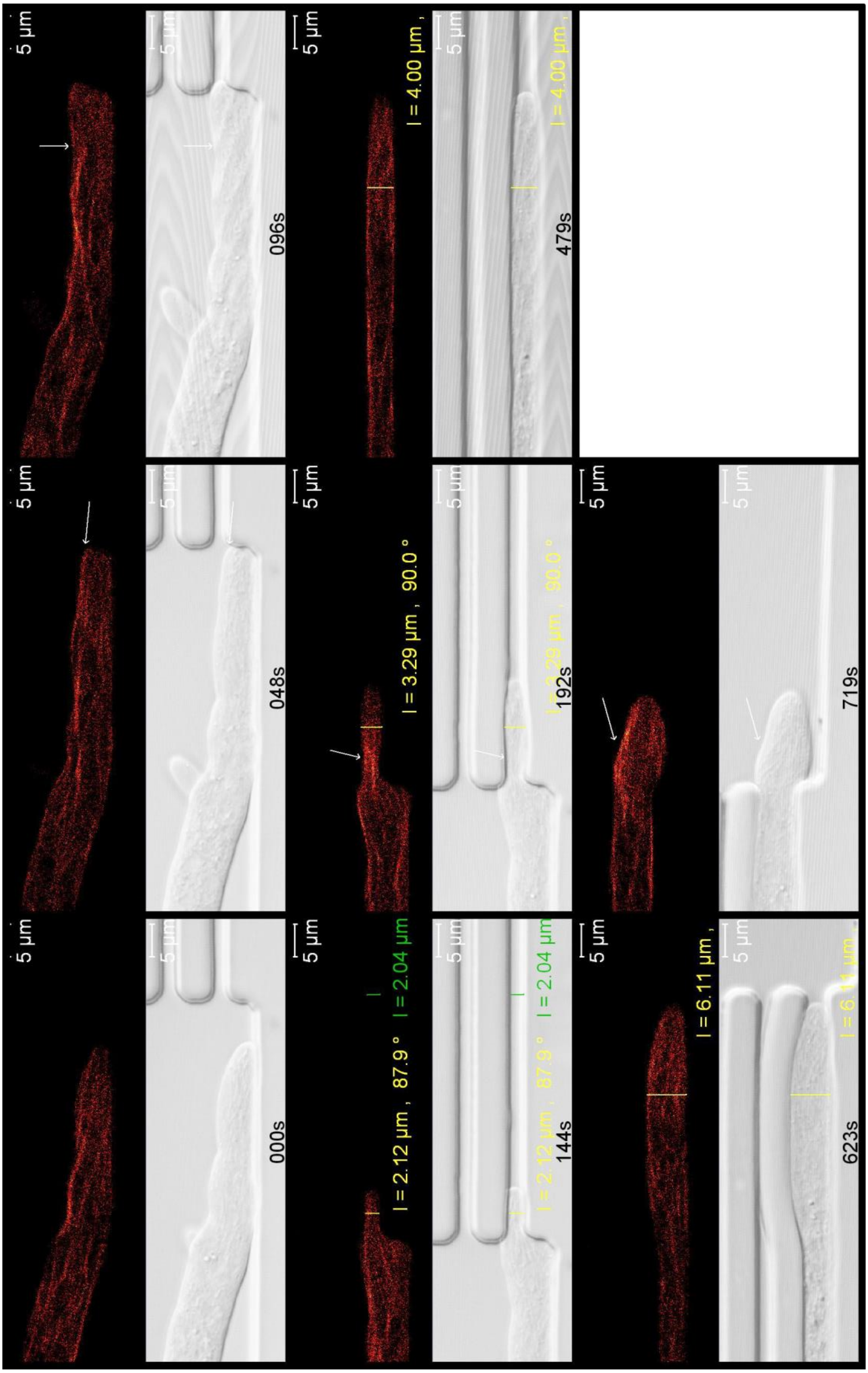

Figure S28. Microtubules in *Neurospora crassa* GFP during the penetration of a 2μm wide channel. The yellow lines indicate the hyphal widths and the green lines indicate the channel width. The arrows indicate specific locations and accumulations of microtubules.

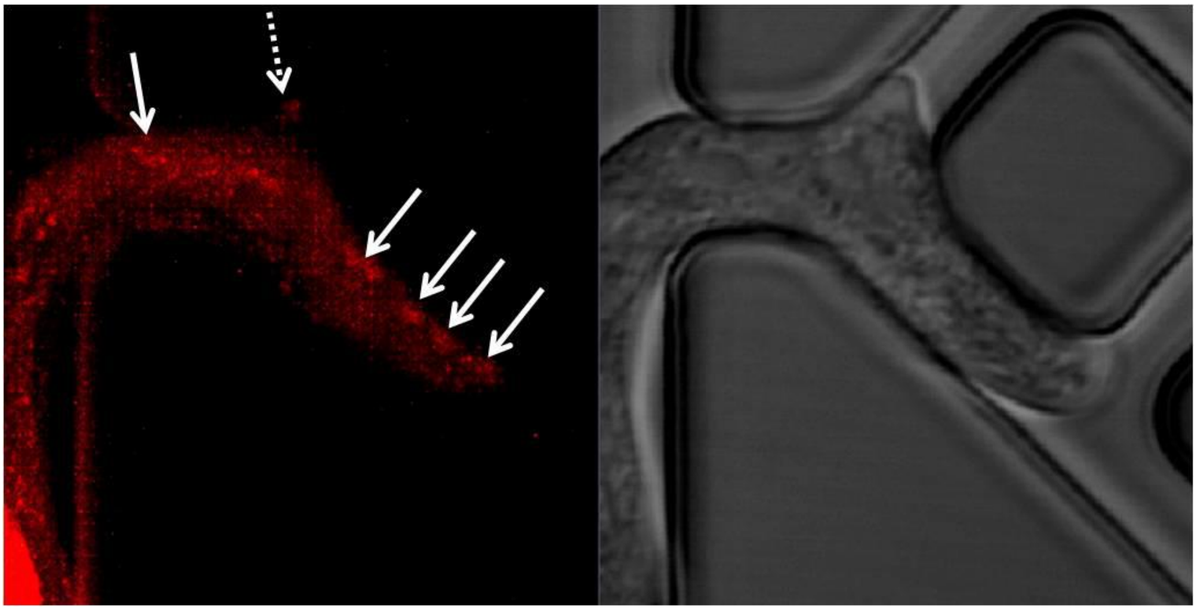

Figure S29. Spitzenkörper mobility and location during branching into channel openings and after bottlenecks. Overlay of the maximum intensities of 121 images of a time series depicting a hypha passing a bottleneck and branching into a channel opening, which was accompanied by the de novo formation of a daughter Spitzenkörper. The images were recorded at intervals of 12.6 s. The parent Spitzenkörper was frequently located close to the confining geometry in the direction closest to the directional memory. The discontinuity appeared to be caused by its motility in the z-direction, which caused it to repeatedly move out of the focal plane. The bright field image represents the hyphal morphology in the last frame of the image series.

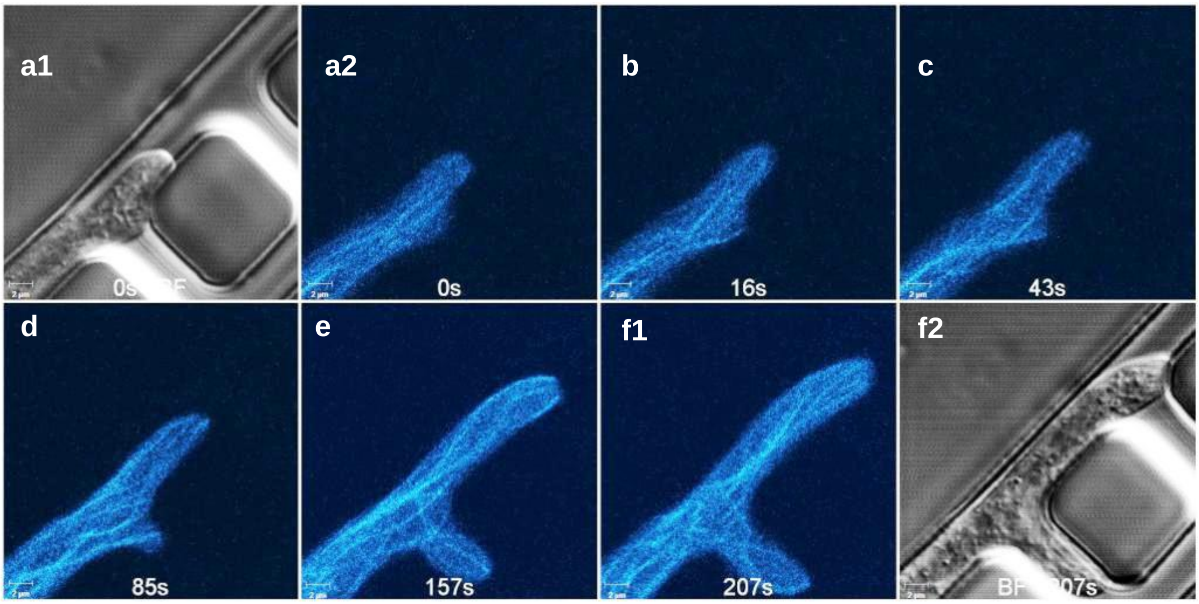

Figure S30. Microtubules in *Neurospora crassa* GFP during branching in a channel with lateral opening. The first and last images represent the simultaneously recorded bright field images of the first and last fluorescence image. The first frame demonstrates the lack of microtubules in the forming bulge. In the following frames, the microtubules penetrated further into the forming daughter branch. Frames a1 and a2, and f1 and f2 represent the same instances.

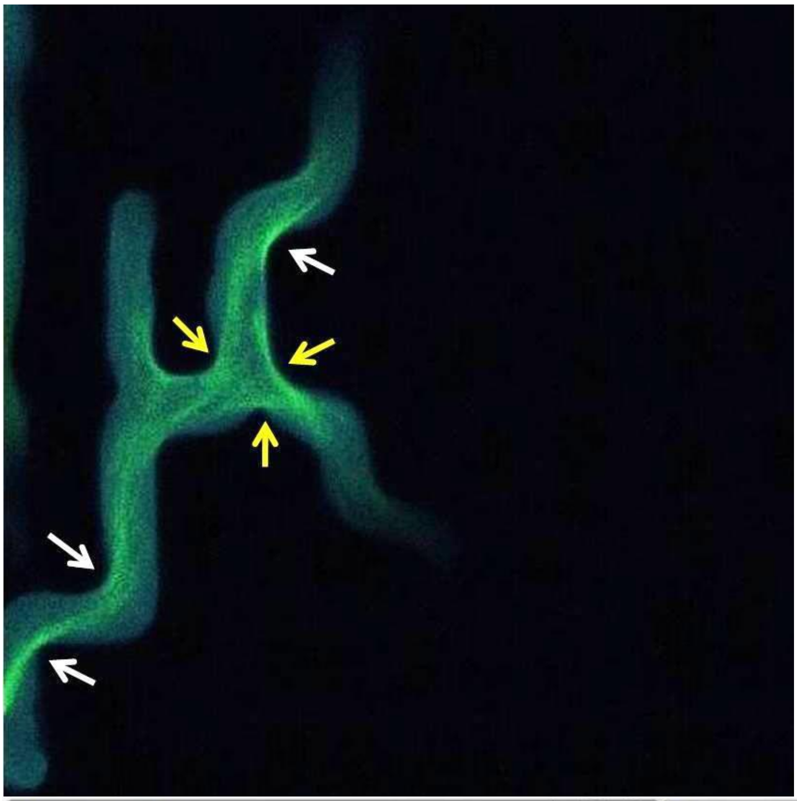

Figure S31. Microtubules in *Neurospora crassa* GFP in meandering channels. The image was created as an average of 51 images in a series recorded at intervals of 15.8 s. The arrows indicate the microtubules that principally aligned along the shortest paths through the hyphae. The yellow arrows indicate a triangle formed by microtubules in the hyphal cross section.

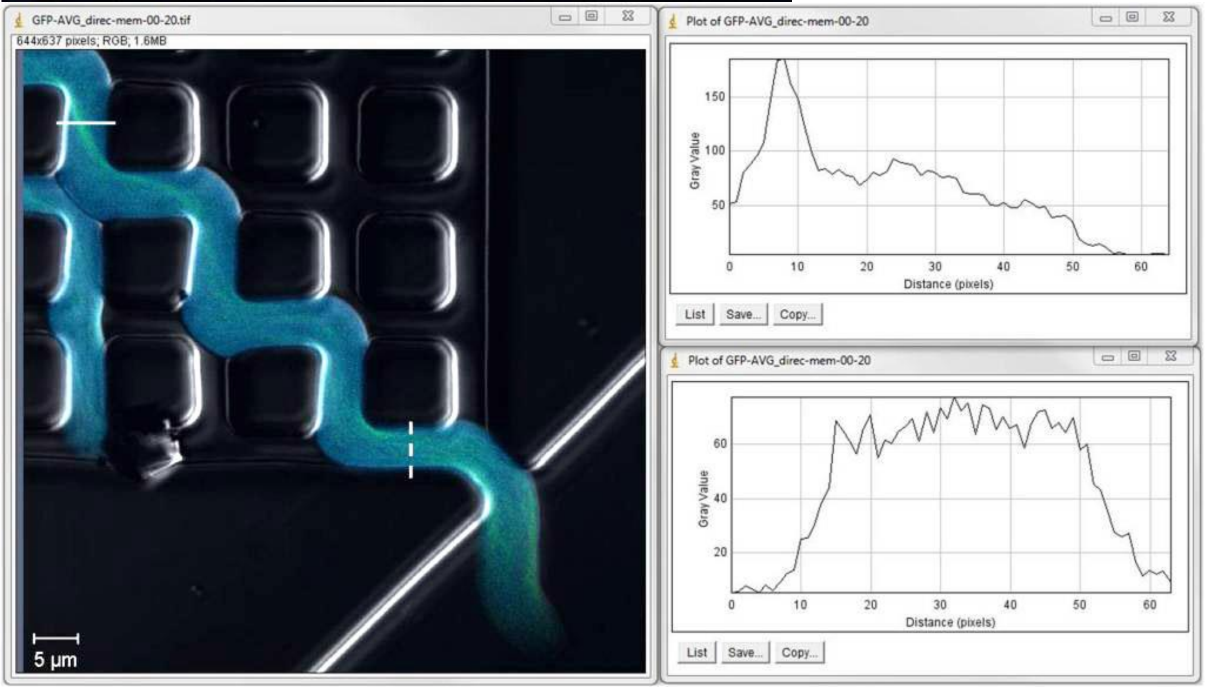

Figure S32. *Neurospora crassa* microtubules through the diamond structure. The average was taken of 21 time-lapse images. The insets represent the intensity profiles taken at the position of the solid white line (top inset) and at the dashed line (bottom inset).

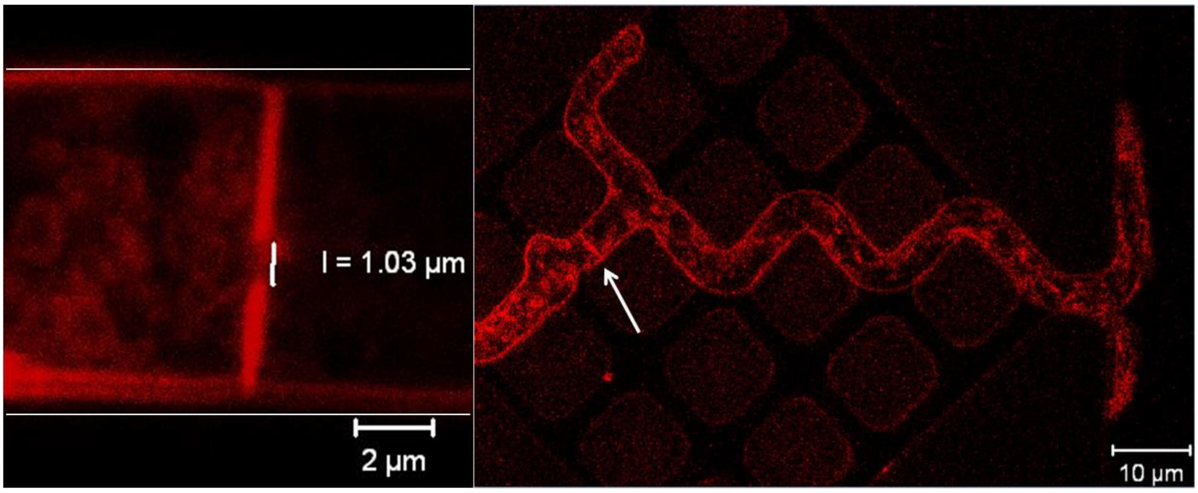

Figure S33. FM4-64 distribution in hyphal trunks confined in channels. Left: Hyphal septum stained with FM4-64 in a hypha confined in a 9 µm wide channel (indicated by white lines). Distribution of the FM4-64 signal in an undulated hyphal trunk in a diamond structure. The white arrow indicates a septum.

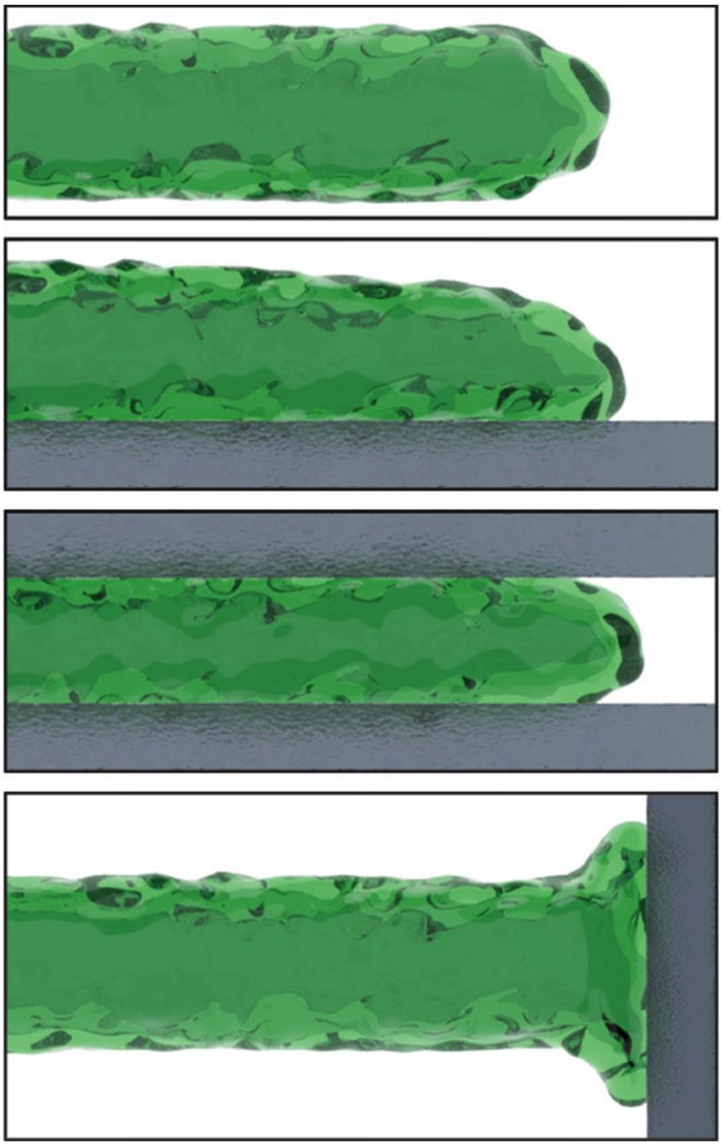

Figure S34. Levels of confinement for fungal growth in PDMS microfluidics structures. (a) *virtually no mechanical confinement:* hyphae with a diameter of 5-7 µm grow in the 10 µm gap between the glass coverslip and the PDMS ‘ceiling’), similar to agar; (b) *parallel 1D confinement*: hyphae progress along a wall in the observation plane; (c) *2D confinement:* hyphae grow while being stretched between two walls that are perpendicular to the observation plane, (d) *orthogonal or angled 1D confinement:* hyphae encounter a wall at near-normal incidence.

1.5. Methods

1.5.1. Microfabrication and experimental setup

The microfluidic network is illustrated in Fig. 1 and see SI Appendix, Fig. S1. Its dimensions, i.e., height of 10 μm, and channel widths ranging from 2 to 100 μm were designed to present various level of containment to fungal growth, from tight-constraining in channels with widths smaller than the hyphal diameter, i.e., 5-7 μm, to confined, but non-constraining chambers, with dimensions of 100 x 100 x 10 μm. The artificial environments were fabricated using a two-component polymer, poly(dimethyldisiloxane) (PDMS, Sylgard 184, Dow Corning) using a well-established procedure.(7) Benefits of using PDMS include low fabrication costs, non-toxicity, good biocompatibility, chemical inertness, and optical transparency for wavelengths as low as 280 nm.(8-12) Briefly, the fabrication involved the moulding of a degassed PDMS mixture of the pre-polymer and curing agent (10:1, w/w) onto a microstructured silicon wafer, at 65°C for a duration in excess of 8 hours. After hydrophilization via exposure to UV/ozone, the PDMS stamps were irreversibly fixed onto a microscope cover slip. Lateral openings in the structure allowed the introduction of the growth medium, fungal hyphae, and fluorescent dyes. Fungal inoculation was achieved by placing an agar plug, extracted from a zone with young hyphae, e.g., the peripheral growth zone of a colony, upside down next to a lateral channel opening. The device was then attached to a microscope slide marked with spacers for accurate positioning on a microscope stage. Hyphal confinement within channels ensured that the hyphae remained within the working distance of the microscope objective, while enabling sufficient gas exchange over long periods of time, thus avoiding the need for perfusion with oxygenated nutrient broth, as required in agar.(13, 14)

The microfluidics network design allowed the investigation of fungal behavior in the following scenarios (see SI Appendix Fig. S33, from top to bottom): (a) *virtually no mechanical confinement*, wherein hyphae with a diameter of 5-7 μm grow in the 10 μm gap between the glass coverslip and the PDMS 'ceiling'), similar to agar; (b) *parallel 1D confinement*, wherein hyphae progress along a wall in the observation plane; (c) *2D confinement*, wherein hyphae grow while being constrained between two walls that are perpendicular to the observation plane; and (d) *orthogonal or angled 1D confinement*, wherein hyphae encounter a wall at near-normal incidence. In many instances, the hyphae encounter the wall at a more acute angle (e.g., 45° or less, relative to the surface), which results in a parallel 1D confinement. Additionally, in the case of 2D confinement, the channels can be given various widths and shapes (e.g., straight, zig-zagged, or bent at various angles).

1.5.2. Fungal species, growth media, staining

The *Neurospora crassa rid (RIP4) mat a his-3+::Pccg-1-Bml+sgfp+* mutant strain (henceforth "*Neurospora crassa GFP*"; FGSC #9519) was obtained from the Fungal Genetics Stock Center (School of Biological Sciences, University of Missouri, Kansas City, MO, USA). The *Neurospora crassa GFP* mutant was constructed(15) to express intrinsically GFP-labelled microtubules while maintaining a growth pattern similar to that of the wild type. The strain was cultured on 1% w/v malt extract agar (Merck), which was also used for medium filling the microfluidics structures. The high level of nutrients ensures the cancelling of the possible chemotaxis-driven directionality of growth. Prior to each experiment, the fungal strains were sub-cultured on fresh malt extract agar plates and incubated at 21°C ± 2°C.

The FM4-64 dye (Invitrogen Ltd. (Paisley, UK) was used as a marker for Spitzenkörper(16). A 20-μl droplet of an 8 μM FM4-64 solution was applied onto a microscope coverslip before placing an agar slab, excised from the margin of the growing colony, upside-down onto the droplet. To avoid an overlay of the dynamics of the dye loading and of the Spitzenkörper, imaging was performed at least one hour after loading the hyphae with the dye.

1.5.3. Time-lapse microscopy and image analysis

Live-cell imaging of hyphal growth was performed with an inverted laser-scanning microscope (Zeiss Axio Observer Z1 with LSM 5 Exciter RGB, Carl Zeiss, Göttingen, Germany) with photomultiplier detectors. Samples were excited with 488 nm and 543 nm lasers, and the emitted light was passed through a bandpass filter (505-530 nm) and a 650 nm long-pass filter. To reduce photobleaching and phototoxic effects, the laser intensity and laser scanning time were kept to a minimum (0.7 - 2.4 % laser energy, 0.75- to 23-second frame scans). Fluorescence and bright-field time-lapse images were captured simultaneously and analyzed using image processing software (Zen 2008, Carl Zeiss, Göttingen, Germany). Fiji software(17) was used for image overlay and quantitative image analysis. RETRAC 2.10.0.5 (from Dr. Nick Carter, University of Warwick, UK) was used for frame-by-frame tracking and calculating cytoskeletal and hyphal kinetics.

1.5.4. Growth experiments on agar and microfluidics structures

Control measurements for fungal growth in non-constraining environments were performed on 1% w/v malt extract media using somatic hyphae at the edges of the colony. The leading hyphae, i.e., wide hyphae showing rapid cytoplasmic flow,(18) rarely entered the microfluidic structures and were therefore omitted. For the somatic hyphae, 'subapical compartments' were characterized by an increased nuclear density approximately 60 µm from the extreme apex. Hyphal growth rates were measured by tracking the position of the extreme hyphal apices in subsequent frames. Fungal growth was recorded for the period needed to observe hyphal behavior from the entry in, to the exit from the microfluidics network of interest, which require approximately 20 minutes for a straight 100 µm channel. However, due to the more convoluted geometries of some microfluidics structures, and the presence of multiple hyphae, in many instances the image recording lasted more than one hour.

To measure the cytoskeletal alignment within hyphae, tangents were fitted manually to microtubules, and the respective local hyphal longitudinal (i.e., polarization) axes and intersection angles were measured. To measure the rates of microtubule polymerization within the apical compartment, and to distinguish this from motility, the positions of individual filament ends were tracked frame-by-frame, following a methodology reported previously.(19)

The parameters of the obstacle-induced apical 'hit & split' included the time elapsed from the impact to the establishment of the daughter hyphae and the maximum size of the formed bulges immediately before the re-establishment of polarized growth. The hyphal diameter was measured at the time of collision with the obstacle. The maximum bulge size was measured by overlaying the frame of collision with the frame in which the growth pattern of the daughter bulges changed to polarized extension and determining the difference in the apical cell wall location on both sides of the hypha.

1.5.5. Statistical analysis

Statistica 7.1 (Statsoft Inc., OK, USA) and GraphPad Prism 6.01 (GraphPad Software Inc., CA, USA) were used for statistical analysis and correlation tests. Statistical analyses included calculating the mean and standard deviation values of parameters measured, i.e., position, alignment with the hyphal axis, polymerization rate for microtubules, times before reappearance of the Spitzenkörper, and hyphal bulge dimensions, over the total number n data points, reported for each instance. Statistical analyses included all accumulated data from at least 20 separate experiments (unless otherwise stated). GraphPad prism was used to perform a Mann-Whitney test comparing the apical and subapical distributions of the microtubule polymerization rates and the microtubule alignments to the polarization axis respectively.

1.6. References for Supplementary Information

1. Adams PA, Sheppard JG, Ridler GM, Ridler PF. New three-parameter empirical extension of the arrhenius equation suitable for the precise evaluation of pseudo-thermodynamic activation parameters in chemical kinetics. Journal of the Chemical Society, Faraday Transactions 1: Physical Chemistry in Condensed Phases. 1978;74.
2. Merkel TC, Bondar VI, Nagai K, Freeman BD, Pinnau I. Gas sorption, diffusion, and permeation in poly(dimethylsiloxane). Journal of Polymer Science Part B: Polymer Physics. 2000;38(3):415-34.
3. Motawia MS, Damager I, Olsen CE, Møller BL, Engelsen SB, Hansen S, et al. Comparative Study of Small Linear and Branched α-Glucans Using Size Exclusion Chromatography and Static and Dynamic Light Scattering#. Biomacromolecules. 2005;6(1):143-51.
4. Hulst AC, Hens HJH, Buitelaar RM, Tramper J. Determination of the effective diffusion coefficient of oxygen in gel materials in relation to gel concentration. Biotechnology Techniques. 1989;3(3):199-204.
5. Warin F, Gekas V, Voirin A, Dejmek P. Sugar diffusivity in agar gel/milk bilayer systems. Journal of Food Science. 1997;62(3):454-6.
6. Merkel TC, Bondar VI, Nagai K, Freeman BD, Pinnau I. Gas sorption, diffusion, and permeation in poly(dimethylsiloxane). Journal of Polymer Science, Part B: Polymer Physics. 2000;38(3):415-34.
7. Held M, Edwards C, Nicolau DV. Probing the growth dynamics of Neurospora crassa with microfluidic structures. Fungal Biology. 2011;115(6):493-505.
8. Desai TA. Micro- and nanoscale structures for tissue engineering constructs. Medical Engineering and Physics. 2000;22:595-606.
9. DiLuzio WR, Turner L, Mayer M, Garstecki P, Weibel DB, Berg HC, et al. Escherichia coli swim on the right-hand side. Nature. 2005;435:1271-4.
10. Mondal S, Ahlawat S, Rau K, Venkataraman V, Koushika SP. Imaging in vivo Neuronal Transport in Genetic Model Organisms Using Microfluidic Devices. Traffic. 2011;12(4):372-85.
11. Park S, Wolanin PM, Yuzbashyan EA, Lin H, Darnton NC, Stock JB, et al. From the Cover: Influence of topology on bacterial social interaction. Proceedings of the National Academy of Sciences. 2003;100(24):13910-5.
12. Kim HJ, Huh D, Hamilton G, Ingber DE. Human gut-on-a-chip inhabited by microbial flora that experiences intestinal peristalsis-like motions and flow. Lab on a Chip. 2012;12(12):2165-74.
13. Löpez-Franco R, Bracker C. Diversity and dynamics of the Spitzenkörper in growing hyphal tips of higher fungi. Protoplasma. 1996;195(1-4):90-111.
14. Riquelme M, Bartnicki-Garcia S. Key differences between lateral and apical branching in hyphae of Neurospora crassa. Fungal Genetics and Biology. 2004;41(9):842-51.
15. Freitag M, Hickey PC, Raju NB, Selker EU, Read ND. GFP as a tool to analyze the organization, dynamics and function of nuclei and microtubules in Neurospora crassa. Fungal Genetics and Biology. 2004;41(10):897-910.
16. Araujo-Palomares CL, Riquelme M, Castro-Longoria E. The polarisome component SPA-2 localizes at the apex of Neurospora crassa and partially colocalizes with the Spitzenkörper. Fungal Genetics and Biology. 2009;46(8):551-63.
17. Schindelin J, Arganda-Carreras I, Frise E, Kaynig V, Longair M, Pietzsch T, et al. Fiji: An open-source platform for biological-image analysis. Nature Methods. 2012;9(7):676-82.
18. Steele GC, Trinci APJ. Morphology and Growth Kinetics of Hyphae of Differentiated and Undifferentiated Mycelia of Neurospora crassa. Journal of General Microbiology. 1975;91(2):362-8.
19. Uchida M, Mouriño-Pérez RR, Freitag M, Bartnicki-García S, Roberson RW. Microtubule dynamics and the role of molecular motors in Neurospora crassa. Fungal Genetics and Biology. 2008;45(5):683-92.

2. Supplementary Movies

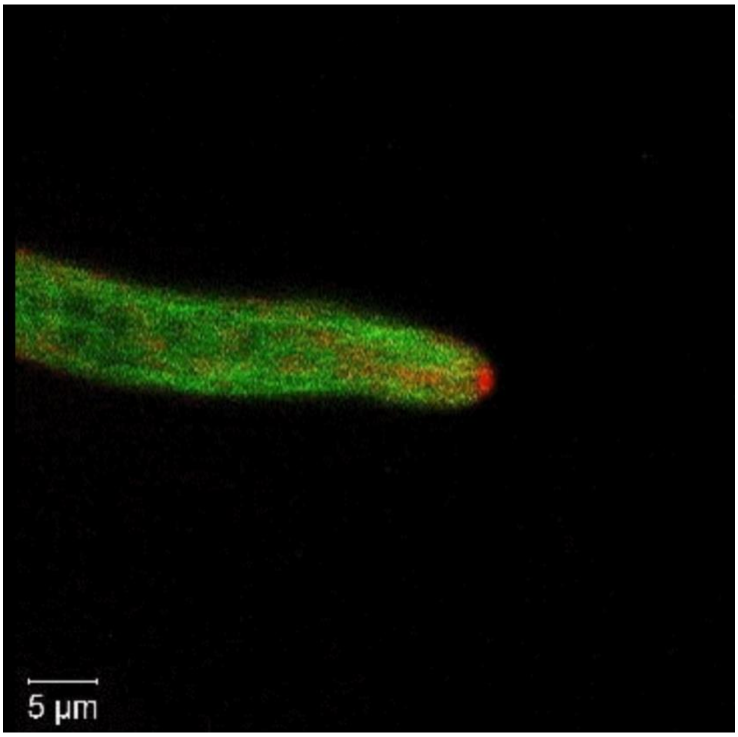

Supporting Information Movie S1. Unconstrained hyphal growth in a wide, 100x100x10 µm PDMS-made chamber. The microtubules were genetically GFP-tagged (pseudo-coloured green), and the hypha was loaded with the marker dye FM4-64 (pseudo-coloured red) labelling the Spitzenkörper as a bright red object within the extreme hyphal apex. The video shows that the Spitzenkörper maintains a central position with temporary side-ways deviations resulting in small changes in the growth direction. Frame rate = 12.6 s per frame.

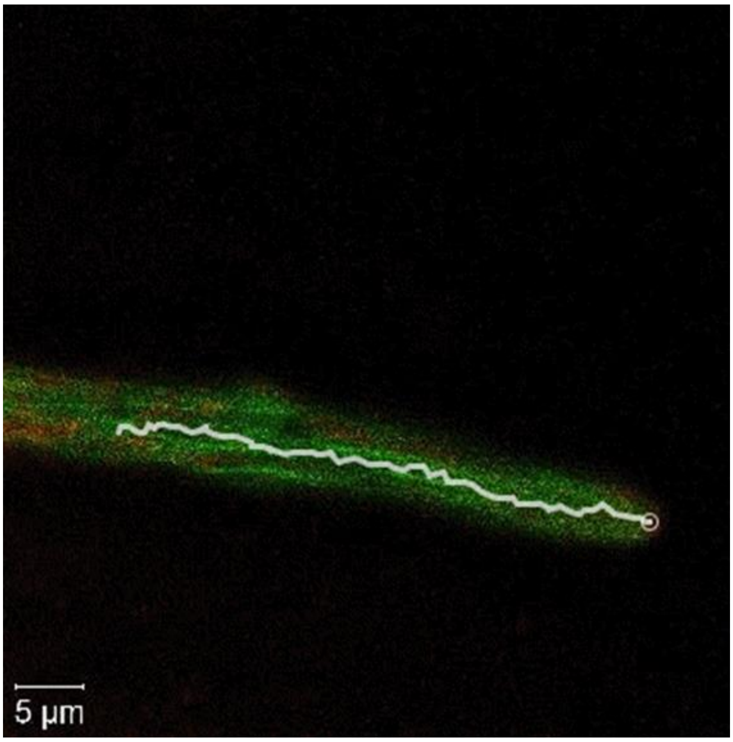

Supporting Information Movie S2: The same hypha as in S1, with tracking of the Spitzenkörper trajectory.

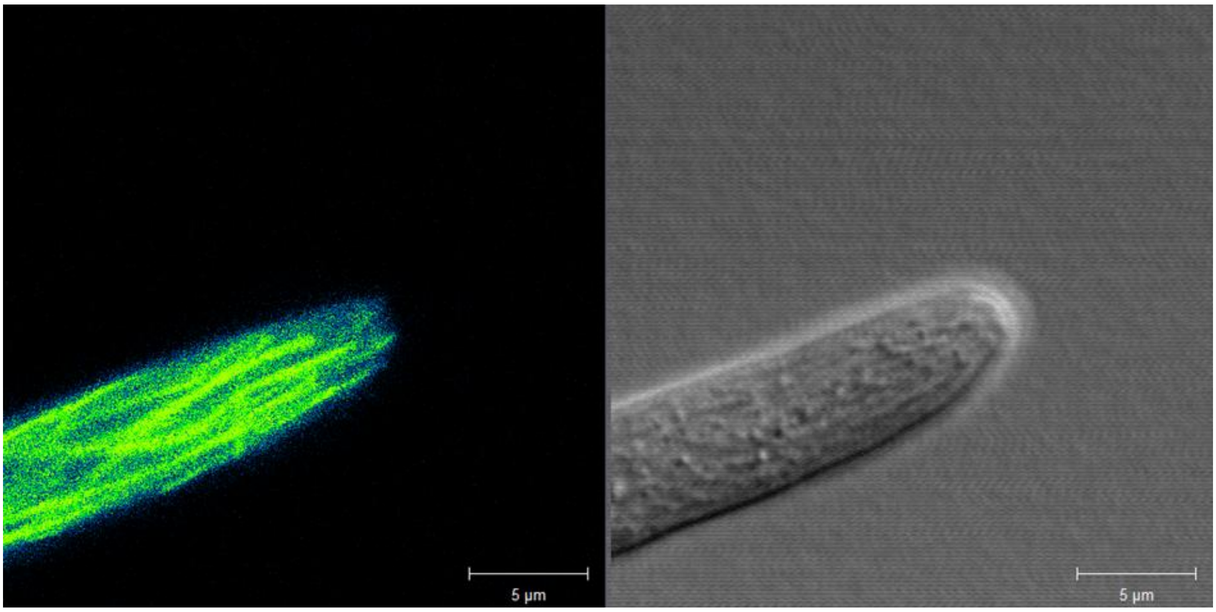

Supplementary Information Movie S3. Hyphal growth on an open agar surface, displaying the microtubules (green, left) and vesicle traffic (phase-contrast image, right).

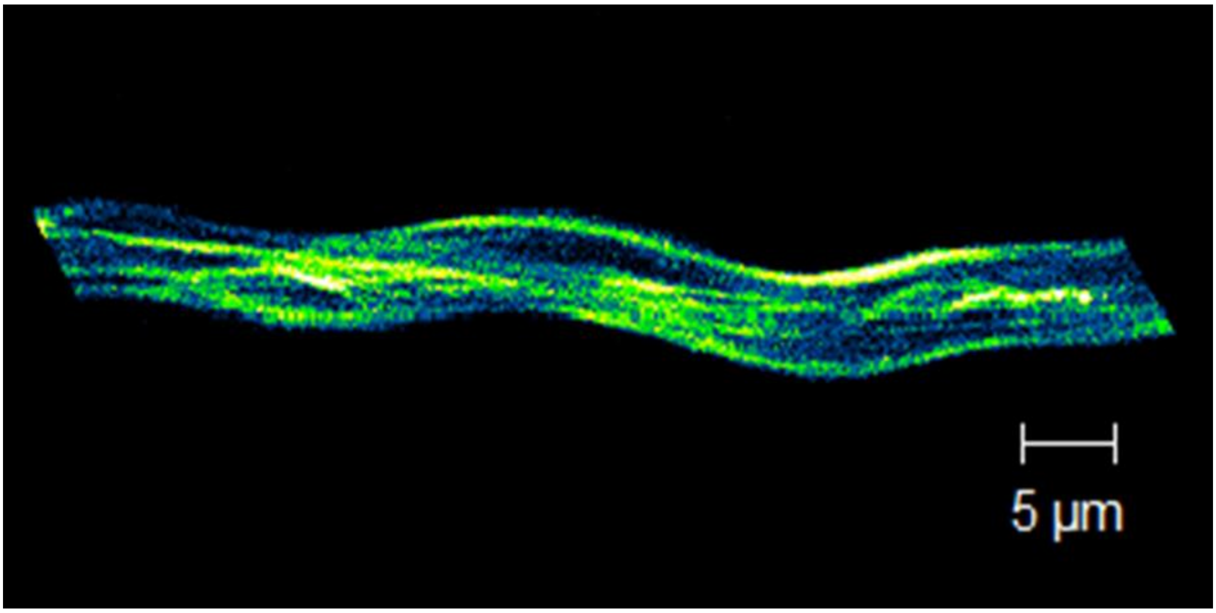

Supporting Information Movie S4: Microtubule distribution along a hypha growing in an unconfined environment.

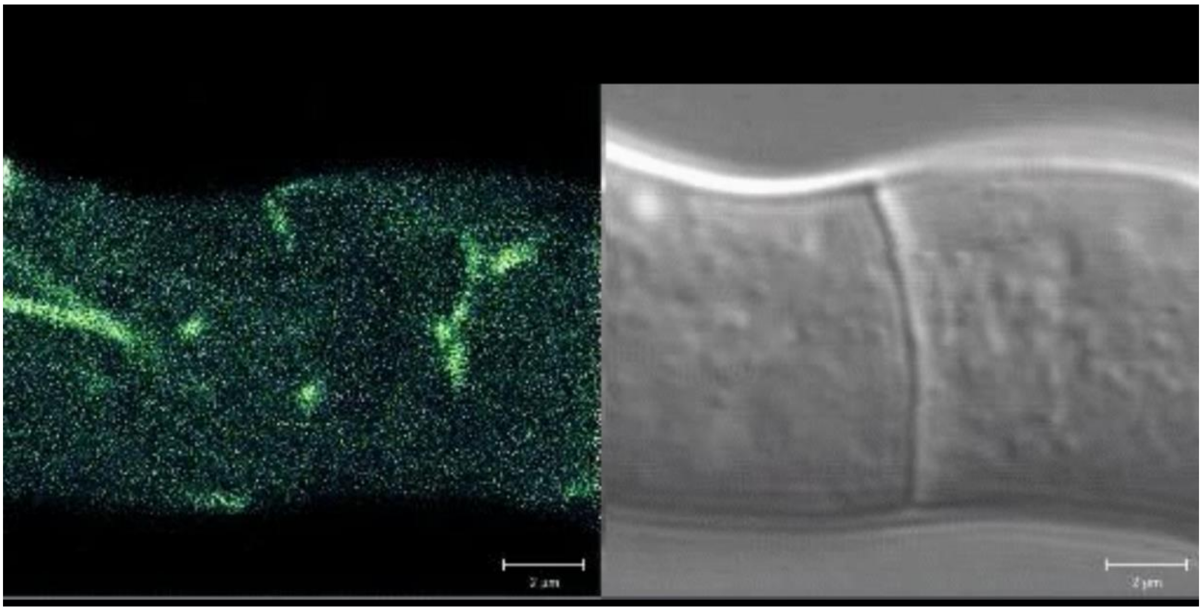

Supplementary Information Movie S5. Microtubule dynamics when passing a septum. Left: fluorescence imaging. Right: bright field imaging.

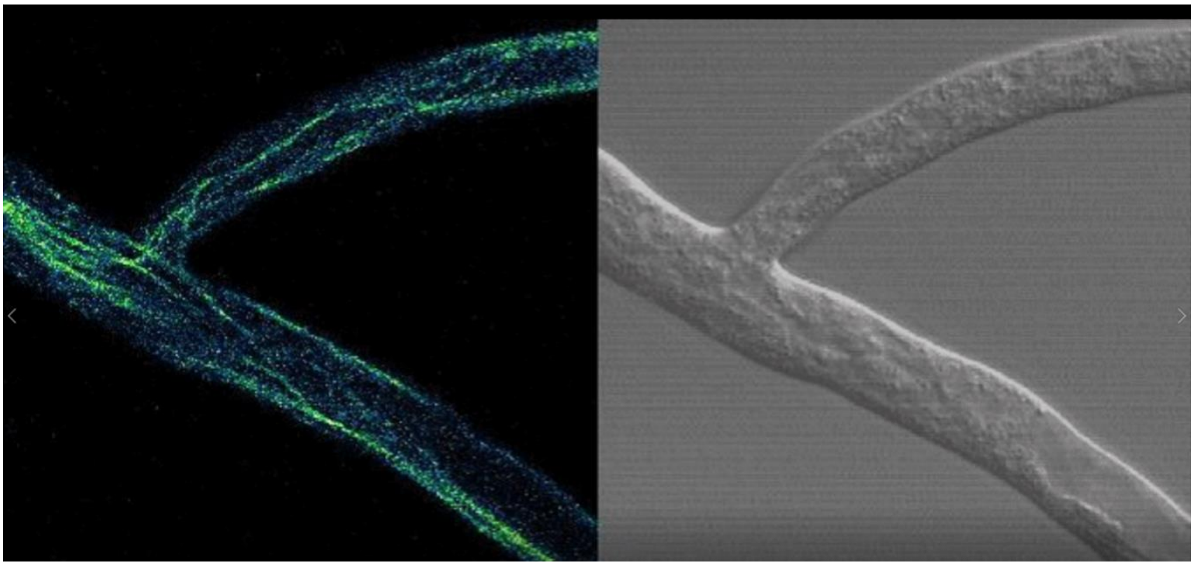

Supporting Information Movie S6: Microtubule dynamics during lateral branching on agar.

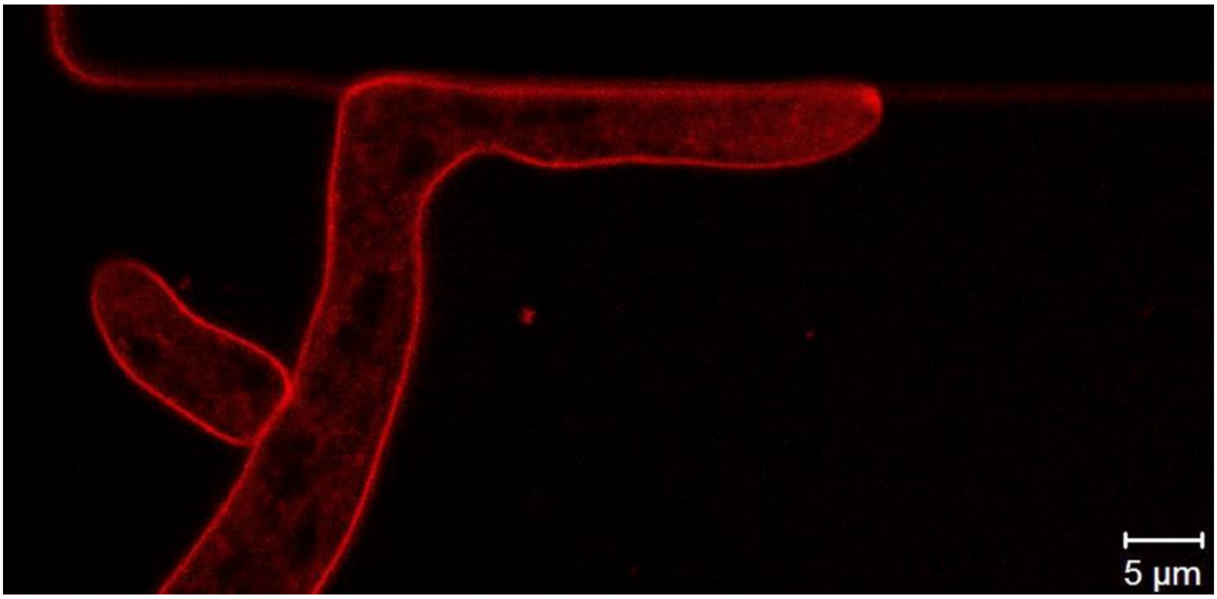

Supporting Information Movie S7. Directional memory, illustrated with a *Neurospora crassa* hypha loaded with the marker dye FM4-64 (pseudo-coloured red). The hypha initially encounters a wall at a steep angle and temporarily redirects its growth direction according to the constraining geometry. During the ‘nestling’ phase, the Spitzenkörper shifts from the apex centre toward the wall, and the apex shape becomes skewed also toward the wall. Upon reaching the corner, the hypha immediately recovers its initial growth direction. Frame rate = 17.9 s per frame; total real-time sequence duration = 20 min 44 s.

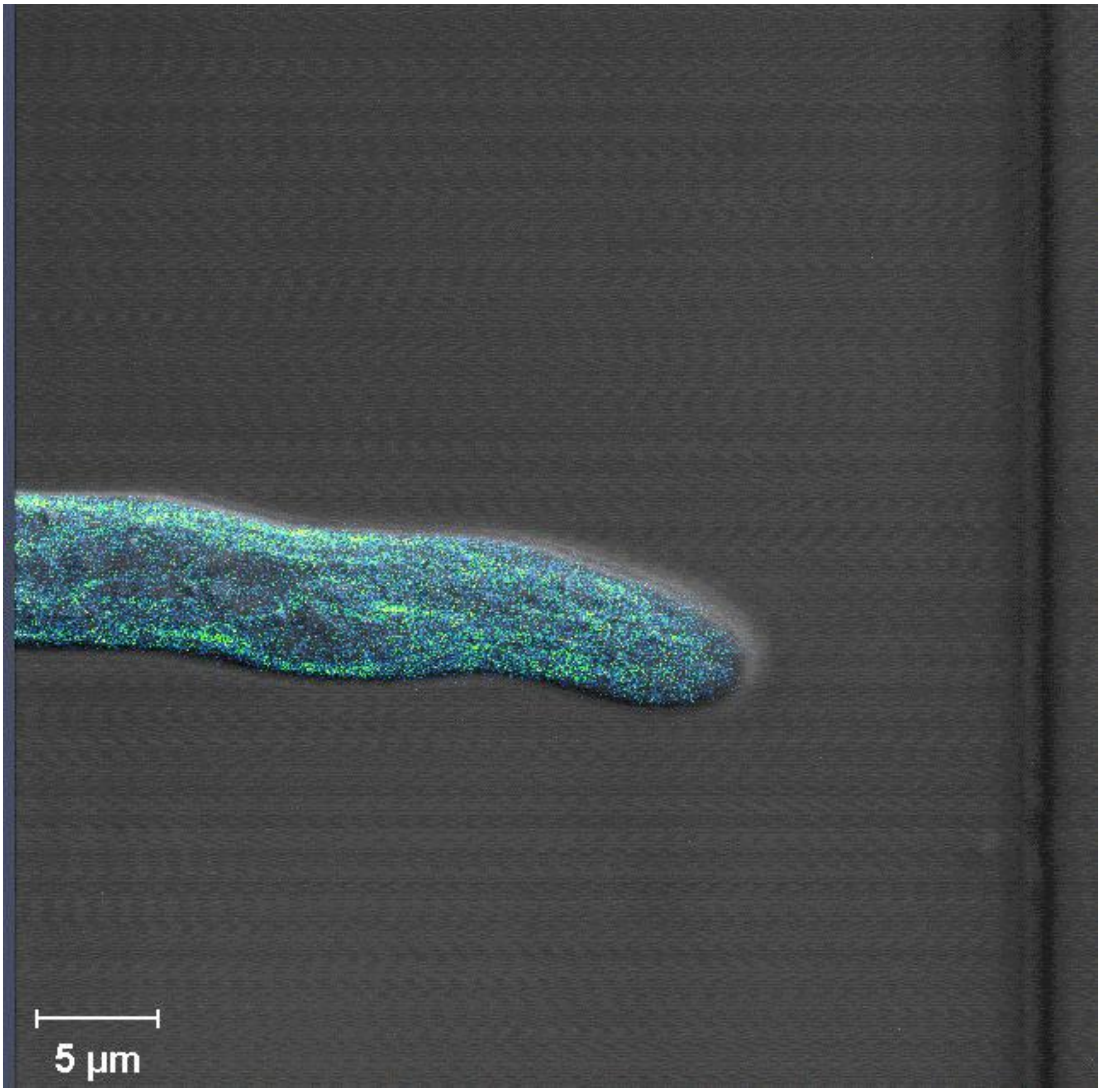

Supplementary Information Movie S8. Encounter of a hypha with a PDMS wall, followed by splitting into two daughter hyphae. After formation the two twin hyphae return to their parent directional memory and penetrate the soft PDMS wall.

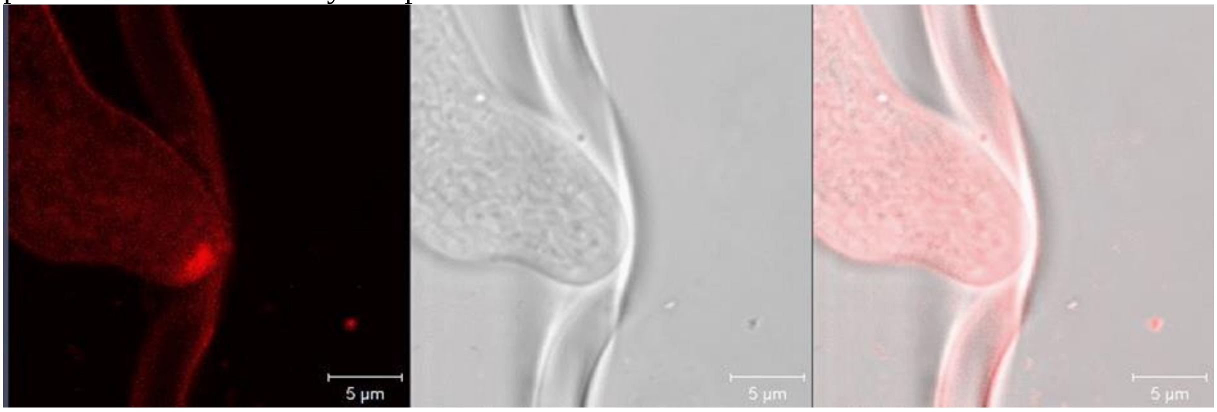

Supplementary Information Movie S9. Encounter of a hypha with a soft PDMS wall, followed by penetration. The Spitzenkörper disappears as the hypha tip exerts pressure on the PDMS wall, similarly with initial stages of the 'hit and split' process (Movie S8).

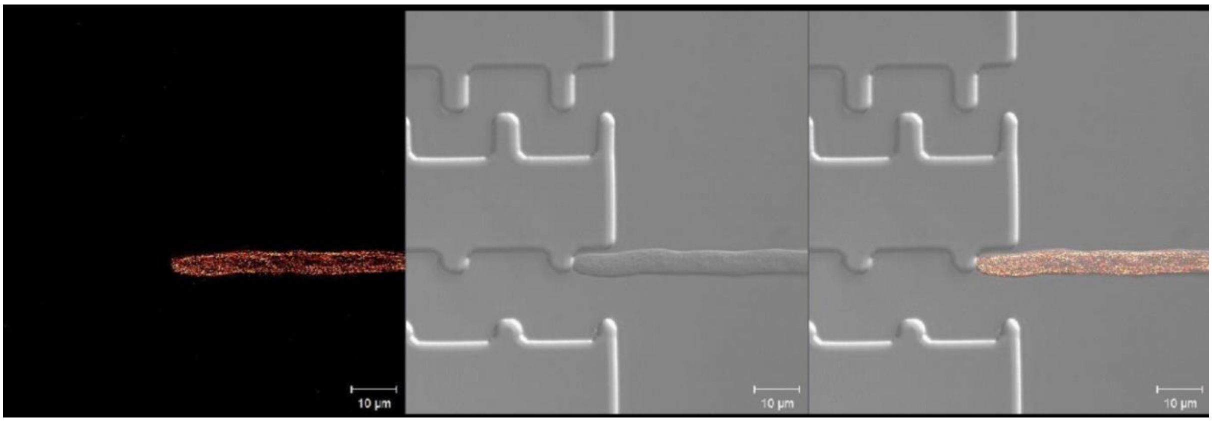

Supplementary Information Movie S10. Microtubule dynamics in a *Neurospora crassa* hypha growing through a string of lateral obstacles. Left: fluorescently labelled microtubules. Middle: differential contrast imaging of the hyphal growth. Right: overlap of the fluorescent and bright field images.

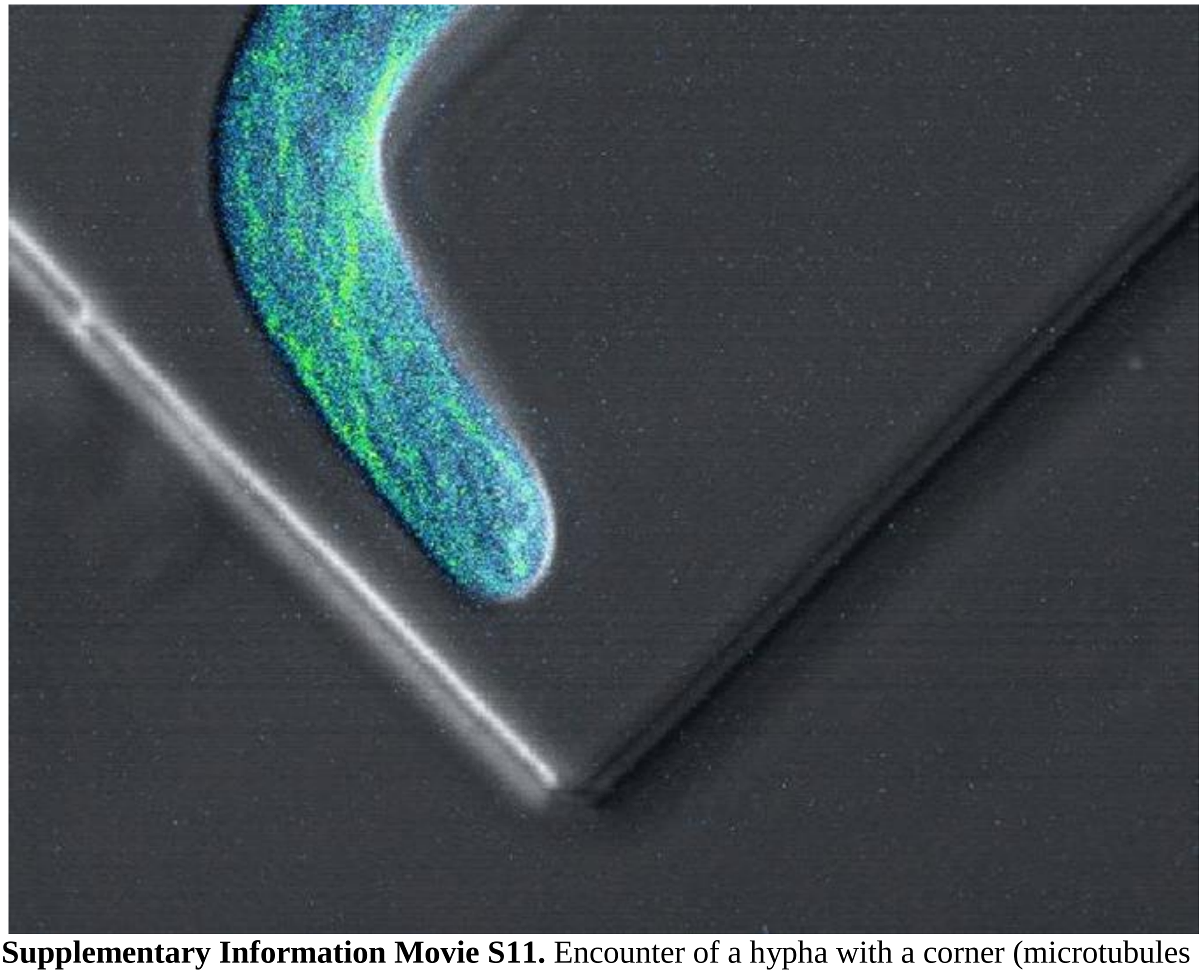

Supplementary Information Movie S11. Encounter of a hypha with a corner (microtubules labelled green). The directional memory opposes a change in growth direction; instead, an orthogonal branch emerges near the apex of the parent hypha.

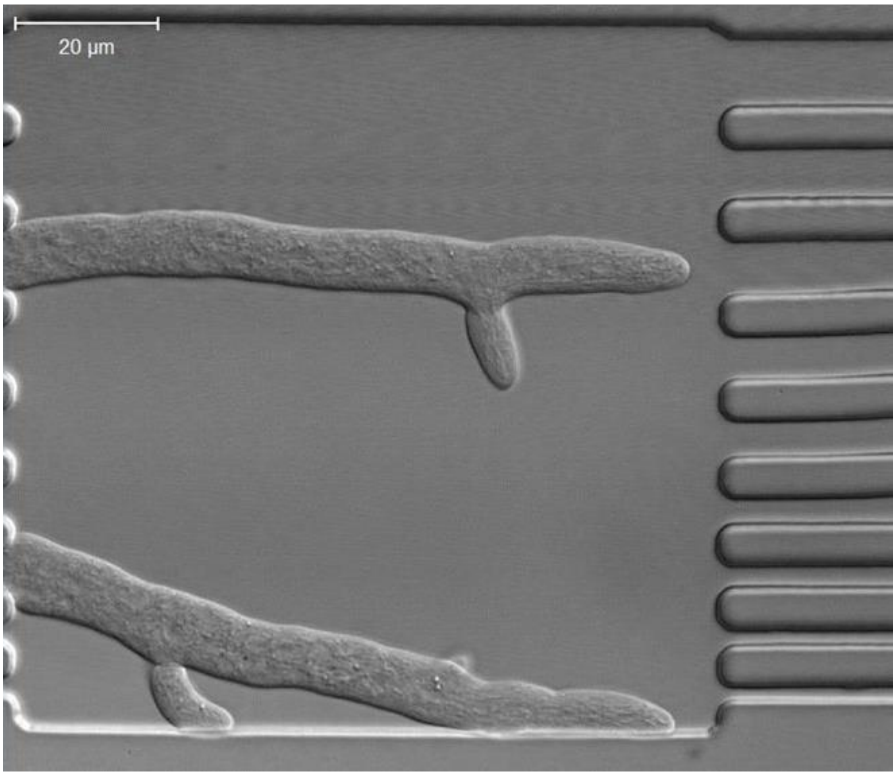

Supplementary Information Movie S12. Hyphae tightly-constrained in narrow channels. The absence of lateral openings precludes lateral branching, but branching resumes immediately upon leaving the channel.

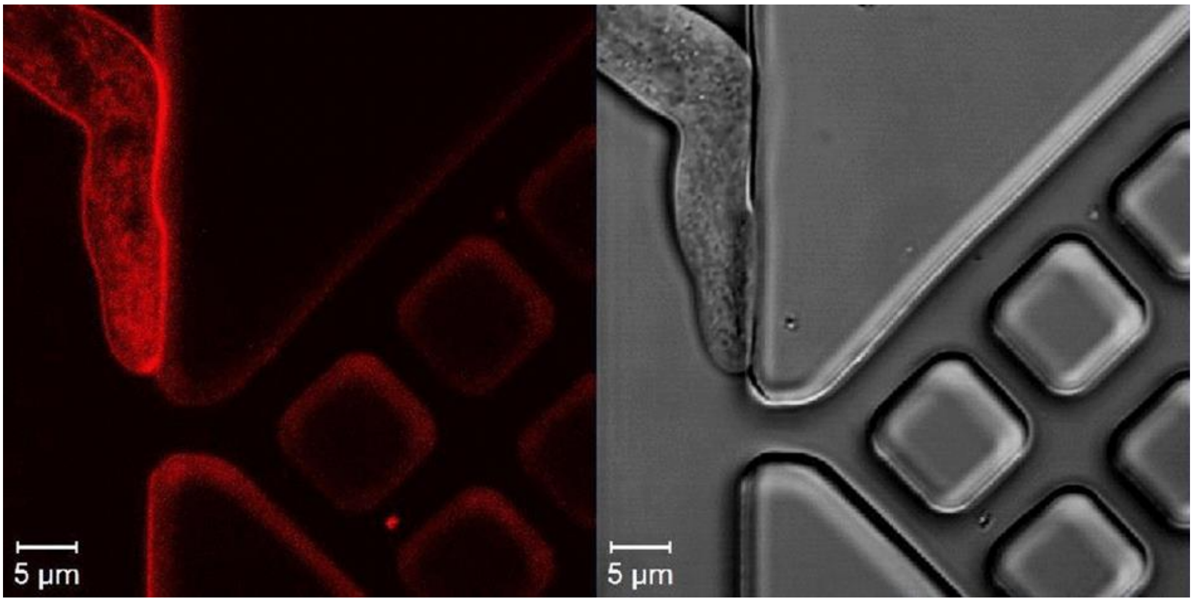

Supplementary Information Movie S13. Lateral branching of a *Neurospora crassa* hypha at a channel intersection, imaged with the fluorescent marker dye FM4-64 (left, pseudo-coloured red) and by bright-field imaging. The growth of the parent hypha is temporarily deflected by the vertical wall, but it subsequently realigns in a direction similar to that before the initial wall encounter (top left corner of the images). During this growth, the expanding subapical hyphal diameter eventually exceeds the channel width at the first intersection. The cell wall bulges, and the formation of a daughter Spitzenkörper precedes the establishment of an independent lateral daughter branch. The effect of directional memory is apparent in the movement of the primary hypha. Frame rate = 17.6 s per frame; total real-time duration 14 min 23 s.

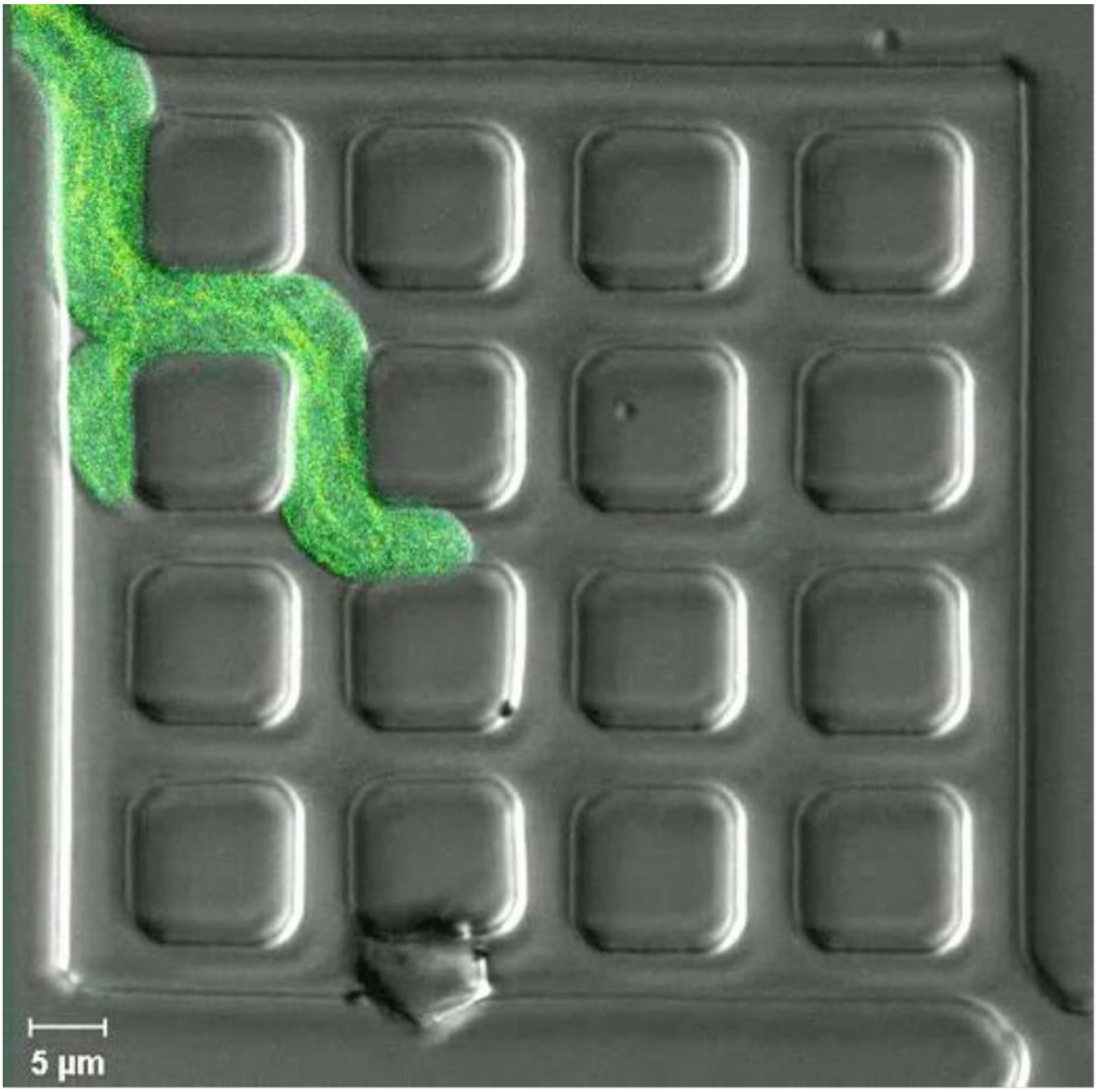

Supplementary Information Movie S14. Directional memory, displayed in the conservation of hyphal microtubule orientations throughout a 150-µm path.

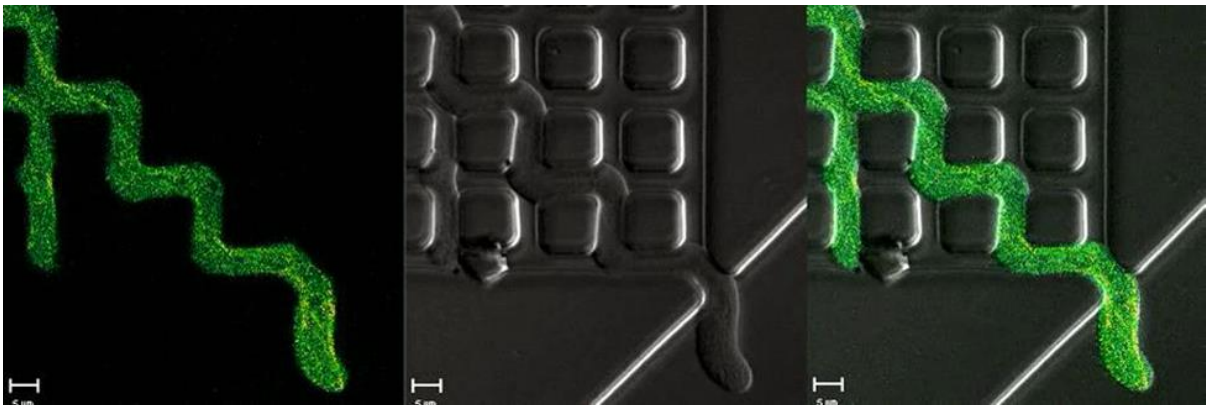

Supplementary Information Movie S15. The same sequence of events as in Movie S9 showing the microtubules only (green, left), a phase-contrast image (middle), and an overlay of the two imaging modes (right).
